# Polyphasic identification of *Rhizopus oryzae* and evaluation of physical fermentation parameters in potato starch processing liquid waste for β-glucan production

**DOI:** 10.1038/s41598-024-66000-5

**Published:** 2024-06-28

**Authors:** Miguel Anchundia, Gualberto León-Revelo, Stalin Santacruz, Freddy Torres

**Affiliations:** 1 School of Food Engineering, Universidad Politécnica Estatal del Carchi, 040101 Tulcán, Ecuador; 2 Faculty of Sciences, Universidad de la República, 11200, Montevideo, Uruguay; 3 School of Agroindustrial Engineering, Universidad Laica Eloy Alfaro de Manabí, 130222 Manta, Ecuador

**Keywords:** β-glucans, *Rhizopus oryzae*, Submerged fermentation, Polysaccharides, Liquid residues from starch processing, Biotechnology, Microbiology

## Abstract

Β-glucans are polysaccharide macromolecules that can be found in the cell walls of molds, such as *Rhizopus oryzae*. They provide functional properties in food systems and have immunomodulatory activity, anticancer, and prebiotic effects; reduce triglycerides and cholesterol; and prevent obesity, among others benefits. Furthermore, potato starch production requires a large amount of water, which is usually discharged into the environment, creating problems in soils and bodies of water. The physical parameters to produce β-glucans were determined, liquid waste from potato starch processing was used and native *Rhizopus oryzae* was isolated and identified from cereal grains. The isolates grew quickly on the three types of agars used at 25 °C and 37 °C, and they did not grow at 45 °C. *Rhizopus oryzae* M10A1 produced the greatest amount of β-glucans after six days of culture at 30 °C, pH 6, a stirring rate of 150 rpm and a fermentation volume of 250 mL. By establishing the physical fermentation parameters and utilizing the liquid waste from potato starch, *Rhizopus oryzae* M10A1 yielded 397.50 mg/100 g of β-glucan was obtained.

## Introduction

Β-glucans are polysaccharide macromolecules formed by D-glucose monomers linked through β-glucoside bonds that can be found in the cell walls of yeasts, molds, bacteria, algae and cereals, which makes their molecular structure and biological activity distinct. The structure of the β-glucans from molds has different molecular structures ranging from α-(1–3) linear bonds to β-(1–6) and β-(1–3) branches^1,2^.

These polysaccharides play an important role in the development, innovation and renovation of food products since they provide functional properties, as well as health benefits for the people who consume them, including immunomodulatory activity, anticancer, and prebiotic effects; the restoration of the microbial balance of the gastrointestinal tract; the maintenance of glucose metabolism, the reduction of triglycerides and cholesterol, the prevention of obesity, among others^3–6^.

Among fungi, β-glucans from edible mushrooms, such as *Agaricus brasiliensis*, *Agaricus biporus*, *Coprinus comatus*, *Laetiporus sulphureus*, *Pleurotus ostreatus*, *Ramaria botrytis*, and *Termitomyces eurhizus*, have been investigated in greater proportions, followed by the yeasts *Sacharomyces cerevisae and Candida albicans* and, to a lesser extent, filamentous molds such as *Aspergillus niger*, *Aspergillus fumigatus, Aspergillus terreus*, *Aspergillus nidulans*, *Aspergillus wentii,* and *Fusarium solani* DO7, in health and human welfare applications^7,8^.

As noted above, few studies have been carried out with filamentous molds in this field, probably because they cause diseases in humans, affect fruit and vegetable crops, contaminate food postharvest, fodder, and animal feed and can even synthesize mycotoxins^9^.

Cell walls from edible filamentous molds with nutritional potential, such as *Aspergillus oryzae*, *Fusarium venenatum*, *Monascus purpureus*, *Neurospora intermedia* and *Rhizopus oryzae,* have been used to produce proteins and may serve as sources of β-glucans. *Aspergillus oryzae* and *Rhizopus oryzae* are safe for use as they are even considered to be probiotics^10^.

The establishment of the physical parameters of fermentation in a liquid state is relevant, as its understanding enables scaling up at pilot and industrial levels. These parameters are specific to each type of fungus used and must therefore be known. Physical parameters such as temperature, stirring speed, pH, and aeration can modify the biocatalysis environment, contributing to morphological characteristics and physiological behavior, and consequently affecting the performance of the bioprocess^11,12^.

On the other hand, the processes of obtaining starch from different vegetable sources require a large amount of water, which is generally discharged into the environment, causing problems in soils and bodies of water because they contain carbohydrates, protein residues and other nutrients. It is therefore necessary to manage starch residues through their use in different biotechnological applications and contribute to sustainable development goals 6, 12, 13, 14 and 15 of the UNESCO 2030 agenda^13,14^. In this sense, the nutrients provided by these residues can be used for the cultivation of *Rhizopus oryzae*.

The waste obtained from the potato starch production process can be used in the production of metabolites and biomass from various types of microorganisms, such as bacteria, molds, and yeasts. These residues have the advantage of providing nutrients, such as potassium, sulfur, phosphorus, sodium, calcium, magnesium, and manganese; vitamins C, B1, B2, B6, and E; and a nitrogen source. The protein content varied between 0.93 and 1.57%, the sugar content between 0.5 and 0.8%, and the fat content approximately 0.2%. This composition makes it useful as a substrate for conducting liquid fermentation processes with fungi and yeasts^15^.

As indicated, the use of these liquid residues can generate some biotechnological benefits, such as being a cheap source of nitrogenous compounds and mineral substances for microorganisms and may help reduce their pollutant load in the environment, offering an alternative to the problem of their utilization^16^.

Due to its low carbohydrate content, it has the disadvantage of providing little organic carbon, which needs to be supplemented to achieve sufficient productivity of cellular biomass and metabolites. Likewise, they may contain toxic glycoalkaloids that can affect the growth of microorganisms and warrant further study^17^.

There are various perspectives on the use of liquid waste obtained from the potato starch extraction process, and several studies have been conducted on this matter.

For the production of biomass of *Candida utilis* and β-glucans, the cultivation of the yeasts *Candida inconspicua*, *Debaryomyces hansenii*, *Kluyveromyces marxianus*, *Kazachstania unispora*, and *Zygotorulaspora florentina*, and the biosynthesis of lipids; the biodegradation and biomass production of the fungal strains *Aspergillus oryzae* 448, *Aspergillus niger* 334, and *Rhizopus oligosporus* 2710; and the production of carotenoids by the yeast *Rhodotorula glutinis*^15,17–20^.

In this study, native *Rhizopus oryzae* were isolated and identified from cereals to determine the physical parameters of fermentation in liquid waste from starch processing to obtain β-glucans from cell walls.

## Results

### Isolation and identification of *Rhizopus oryzae*

Based on the initial characteristics of the cottony texture and the white, light gray, and dark gray colors of the colonies, 19 presumptive isolates of *Rhizopus oryzae* were obtained and separated into three groups based on growth temperatures of 37 and 45 °C in MEA, PDA and OA media. Group I grew at both temperatures and consisted of the isolates M4A2 and M4A9; group II did not grow at 37 and 45 °C and consisted of the isolated M4A1, M4A5-M4A8, M4A10-M4A12, M8A1 and M10A3; and group III grew at 37 °C and did not grow at 45 °C and consisted of the isolated M4A3, M4A4, M7A1-M7A3 and M10A1.

The characteristics of the group I isolates in MEA grown at 25 °C are shown in Supplementary Fig. S1, S2 and Supplementary Table S1. The color of the group I isolates was dark gray in the center with a white edge, and the size of the colonies ranged between 2.00 and 9.00 cm. In MEA, PDA and OA, the growth rate was medium to fast for the M4A2 isolate and slow to fast for the M4A9 isolate, and the margin was entire and wavy for both isolates.

The isolates of group II presented varied colors; in the center of the colonies, white, light gray and dark gray colors were observed, while the edges were white, dark gray and black.

**Figure 1:**
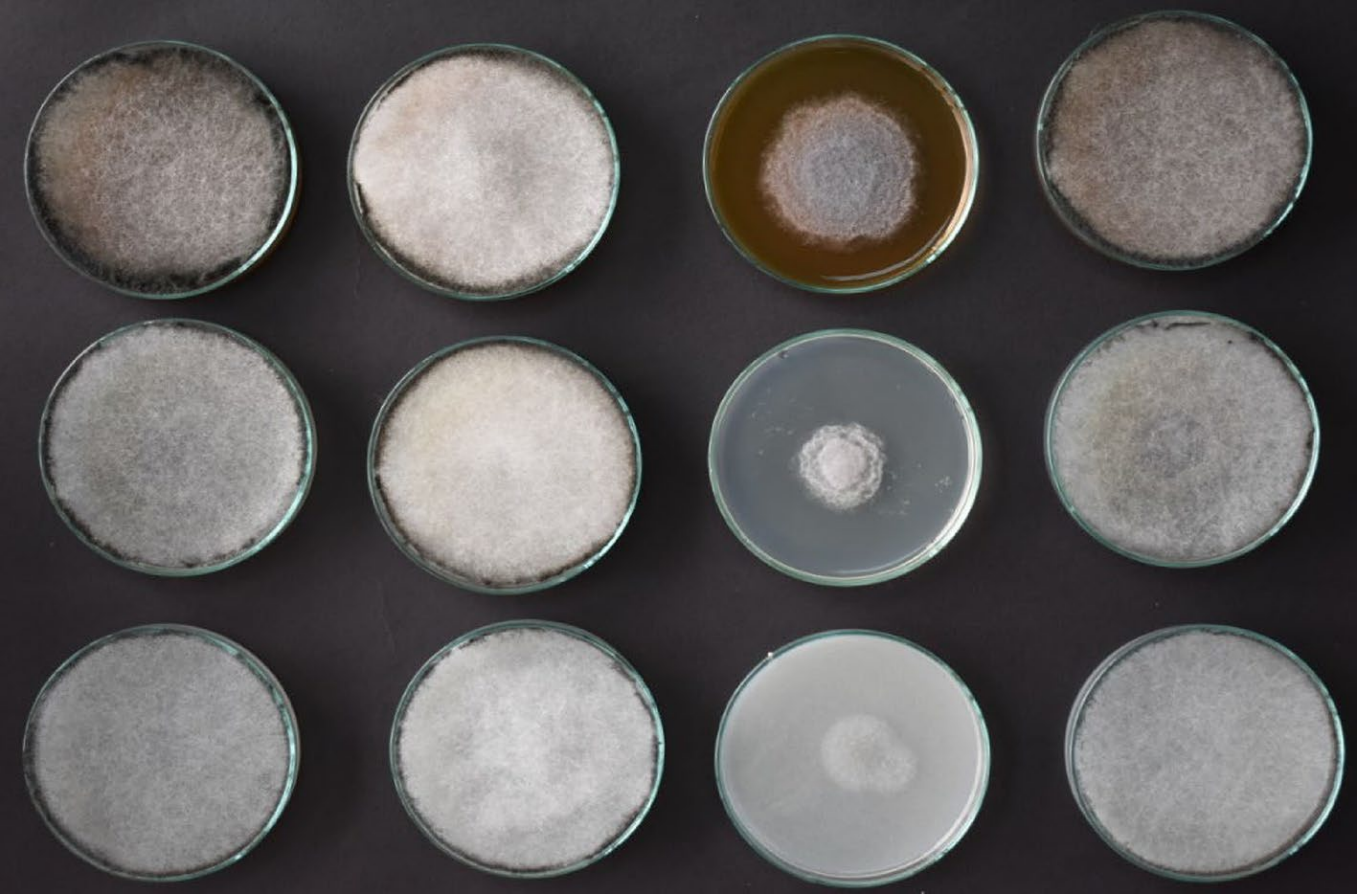
Colonies of presumptive isolates of *Rhizopus oryzae* grown at 25 °C for 4 days. Column 1 = M8A1, column 2 = M10A1, column 3 = M10A2 and column 4 = M10A3. Row 1 shows the MEA isolates, row 2 shows the PDA isolates, and row 3 shows the OA isolates.

At a temperature of 25 °C, in MEA, the colonies grew at a fast rate, in PDA and OA they grew at a medium to fast rate, the margin was entire, and in isolate M10A3, it was wavy (Fig. 1, Supplementary Fig. S1–S3, and Supplementary Table S2).

**Figure 2:**
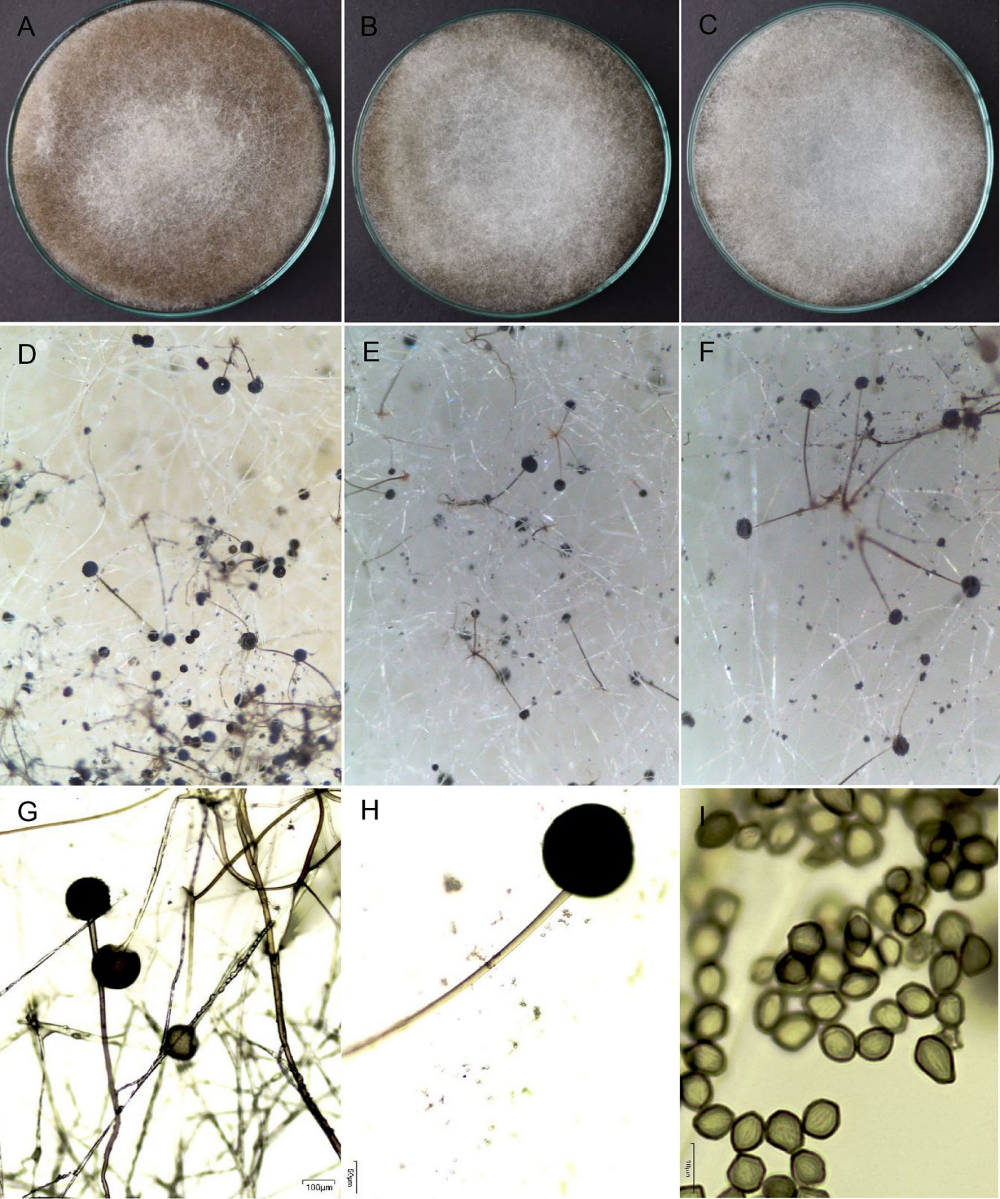
Colonies of the M10A1 isolate of presumptive *Rhizopus oryzae*. A–C. Colonies were grown on MEA, PDA and OA at 37 °C for 4 days. D-E. Texture of colonies cultivated on MEA, PDA and OA at 25 °C for 4 days. F–H. Sporangiophores and sporangiospores were grown on MEA agar at 25 °C. G bar, 100 µm. H bar, 50 µm. I bar, 10 µm.

The characteristics of the group III isolates are presented in Figs. 1, 2, Table 1, Supplementary Figs. S3–S6 and Supplementary Tables S3–S5. At 25 °C in MEA, the color at the edge of the colonies ranged from white to dark gray, while that at the center ranged from light gray to dark gray.

**Table 1:** Macroscopic characteristics of M7A3 and M10A1 isolates cultivated on MEA, PDA and OA at 25 °C, 37 °C and 45 °C for 4 days.

Sample	Agar	Temperature (°C)	Surface color	Back color	Colony size (cm)	Margin	Growth rate	Texture
M7A3 Wheat	MEA	25	Light gray	Cream	9.00 ± 0.00	Entire	Fast	Cottony
		37	Light gray	Cream	9.00 ± 0.00	Entire	Fast	Cottony
		45	There was no growth					
	PDA	25	White	White	9.00 ± 0.00	Entire	Fast	Cottony
		37	Light gray	White	9.00 ± 0.00	Entire	Fast	Cottony
		45	There was no growth					
	OA	25	White	White	9.00 ± 0.00	Entire	Fast	Cottony
		37	White	White	9.00 ± 0.00	Entire	Fast	Cottony
		45	There was no growth					
M10A1 Wheat	MEA	25	Light gray in the center, dark gray in the edge	Orange	9.00 ± 0.00	Entire	Fast	Cottony
		37	Light gray in the center, brown in the edge	Orange	9.00 ± 0.00	Entire	Fast	Cottony
		45	There was no growth					
	PDA	25	Light gray in the center, dark gray in the edge	White	9.00 ± 0.00	Entire	Fast	Cottony
		37	Light gray in the center, dark gray in the edge	White	9.00 ± 0.00	Entire	Fast	Cottony
		45	There was no growth					
	OA	25	Light gray in the center, dark gray in the edge	White	9.00 ± 0.00	Entire	Fast	Cottony
		37	Light gray in the center, dark gray in the edge	White	9.00 ± 0.00	Entire	Fast	Cottony
		45	There was no growth					

The growth rates of M4A3 and M4A4 were intermediate to fast on MEA and PDA and slow on OA for M4A4, whereas those of M7A1-M7A3 and M10A1 grew quickly on all three types of agar. The margins were the same for all the isolates. The isolates that grew at 37 °C, did not grow at 45 °C and showed macroscopic characteristics of *Rhizopus oryzae* were M7A1-M7A3 and M10A1.

The microscopic characteristics of these isolates are presented in Fig. 2A–I and Table 2. The number of sporangia in the isolates ranged between 2 and 3, and these structures had diameters between 49.20 and 130.80 µm. The sporangiophores were between 223.20 and 2890.40 µm long and between 4.70 and 18.60 in diameter. Columella were observed in the isolated M10A1, which had a diameter of 7.50–10.00 µm, and chlamydospores were detected in the isolated M7A1, which had a size of 10.00 × 8.00 µm, 15.20 × 10.30 µm (length × width) and were absent in the other isolates. The spores were elongated with stretch marks.

**Table 2:** Microscopic characteristics of M7A1, M7A2, M7A3 and M10A1 isolates in MEA grown at 25 °C for 4 days.

Microscopic characteristics	Sample identification			
	M7A1	M7A2	M7A3	M10A1
Number of sporangia per group	2	3	3	2
Sporangiophore length (µm)	249.20–2894.40	223.20–823.80	262.30–650.70	1003.30–1862.60
Sporangiophore diameter (µm)	8.00–18.60	4.70–12.80	4.70–13.00	7.50–9.80
Columella diameter (µm)	–	–	–	7.50–10.00
Sporangium diameter (µm)	76.4–81.8	91.00–130.80	49.20–115.50	104.90–118.90
Existence of chlamydospores	Yes	No	No	No
Size of existing chlamydospores (width × length) (µm)	10.00 × 8.00–15.20 × 10.30	–	–	No
Spore size (µm)	8.10–10.30	10.20–26.60	6.50–9.00	6.20–10.70
Spore ornamentation	Elongated with stretch marks	Elongated with stretch marks	Elongated with stretch marks	Elongated with stretch marks

**Figure 3:**
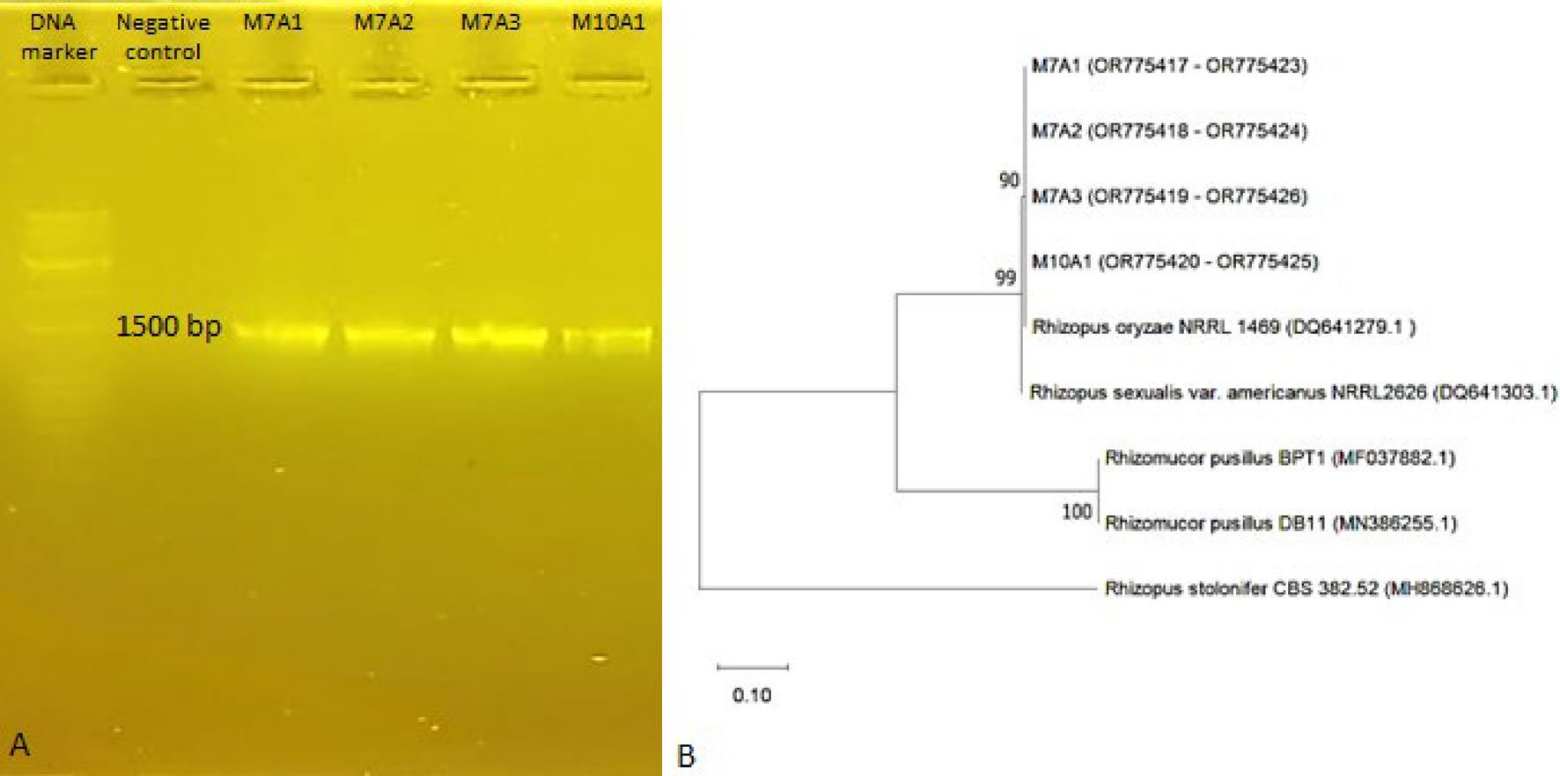
(**A**) Agarose gel electrophoresis of the V9G and LR3 regions of presumptive *Rhizopus oryzae* isolates. (**B**) Phylogenetic tree based on the neighbor joining method of sequences obtained for the ITS1-5.8S-ITS2 region of isolates of *Rhizopus oryzae* species and NCBI reference strains. *Rhizopus stolonifer* was used as an outgroup. The probability values of the nodes for each clade indicate the percentage of the analysis replicas of 1000 bootstraps supporting that clade.

Regarding molecular identification, the amplicon size generated by the primers V9G and LR3 was 1500 bp (Fig. 3A). This region included the entire ITS1-5.8S-ITS2 region and the D1/D2 region of the 28S (LSU) ribosomal and consensus sequences of the ITS1-5.8S-ITS2 region, which were approximately 600 bp.

The phylogenetic tree based on the neighbor joining method with the sequences ITS1 and ITS4 concatenated from the M7A1, M7A2, M7A3 and M10A1 isolates showed that they aligned in a clade defined with the strain type *Rhizopus oryzae* NRRL 1469, NCBI accession number DQ541279.1 (Fig. 3B).

The grouping formed was supported with a 90% bootstrap value. The similarity shown by the isolates M7A1, M7A2 and M10A1 was 100%, and that of M7A3 was 99.62%, which corroborates the macroscopic and microscopic characteristics typical of *Rhizopus oryzae*; consequently, the four isolates were classified with this fungus species.

### Determination of the fermentation parameters

Table 3 shows the results for the characterization of the liquid waste from potato starch processing used as a culture medium for *Rhizopus oryzae*. The component with the highest concentration was starch (3120.30 mg/100 mL of liquid waste). The total solids content was the second most important component of the medium (2.29 g/100 g liquid waste), and the lowest concentration was the ash content with 0.04 g/100 g liquid waste.

**Table 3:** Characterization of liquid residue from potato starch processing.

Components	Content
Total solids (g/100 mL)	2.29 ± 0.04
Soluble solids (g/100 mL)	1.67 ± 0.05
Solids in suspension (g/100 mL)	0.62 ± 0.02
Nitrogen (g/100 mL)	0.15 ± 0.00
Proteins (g/100 mL)	0.96 ± 0.04
Ashes (g/100 mL)	0.04 ± 0.00
Reducing sugars (mg/100 mL)	447.04 ± 43.38
Total sugars (mg/100 mL)	1110.72 ± 17.87
Starch (mg/100 mL)	3120.30 ± 13.29

**Figure 4:**
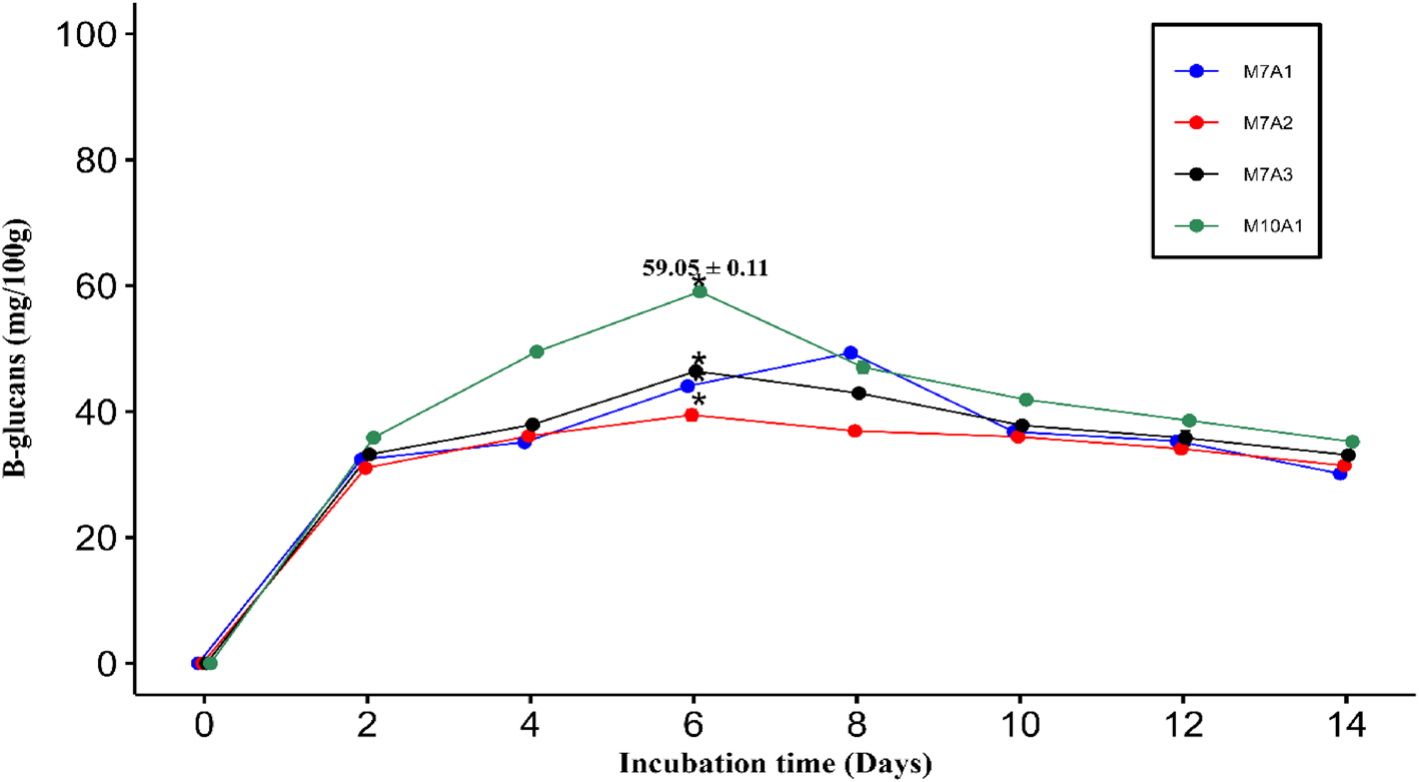
B-glucan production over time by *Rhizopus oryzae* M7A1, M7A2, M7A3 and M10A1.

The effect of time on the culture of *Rhizopus oryzae* in liquid residues is shown in Fig. 4. The maximum amount of β-glucans was reached after six days of culture for M7A2, M7A3 and M10A1 and after eight days for M7A1. After this time, the amount of β-glucans decreased until the lowest value was obtained at 14 days of culture. There were statistically significant differences for the first 3 *Rhizopus oryzae* isolates (*p* < 0.05) at six days of culture, and *Rhizopus oryzae* M10A1 (59.05 mg/100 g of biomass) had the highest polysaccharide productivity.

**Figure 5:**
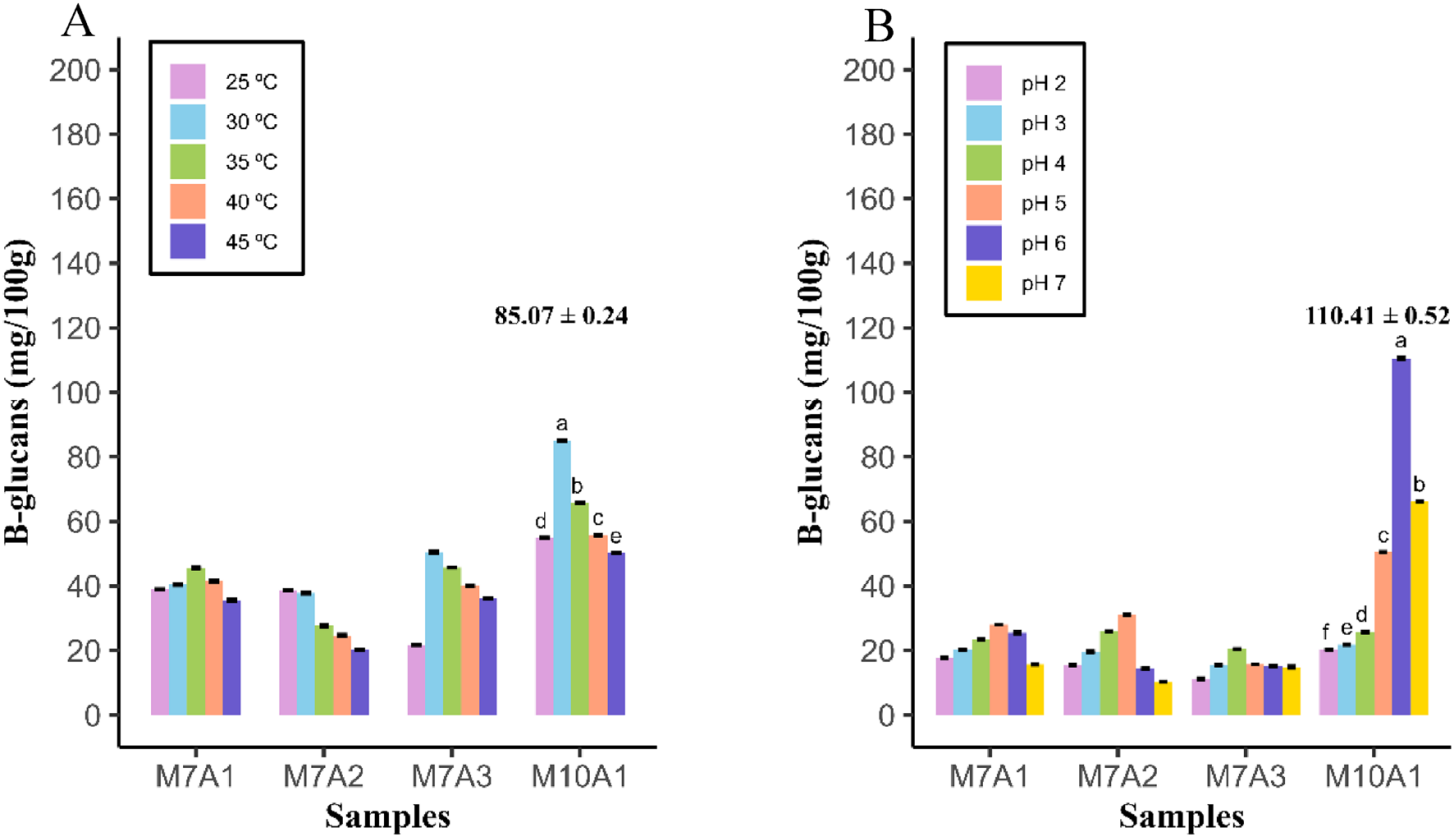
(**A**) β-glucans production at different temperatures by isolates M7A1, M7A2, M7A3 and M10A1. (**B**) Production of β-glucans at different pH values by *Rhizopus oryzae* M7A1, M7A2, M7A3 and M10A1.

Regarding the growth temperature, M7A2 produced the lowest concentration of β-glucans at all tested temperatures, with values between 38.79 and 20.19 mg/100 g biomass at 25 °C and 45 °C, respectively. *Rhizopus oryzae* M10A1 showed a greater polysaccharide yield at 30 °C, producing 85.07 mg/100 g of biomass compared to the other temperatures (*p* < 0.05) (Fig. 5A).

The effect of pH on the *Rhizopus oryzae* culture is shown in Fig. 5B, M7A3 produced the lowest amount of β-glucans at pH 2 (11.30 mg/100 g biomass) and 20.45 mg/100 g biomass at pH 4, while M10A1 produced the greatest amount of β-glucans at pH 6 (110.41 mg/100 g biomass) (*p* < 0.05).

**Figure 6:**
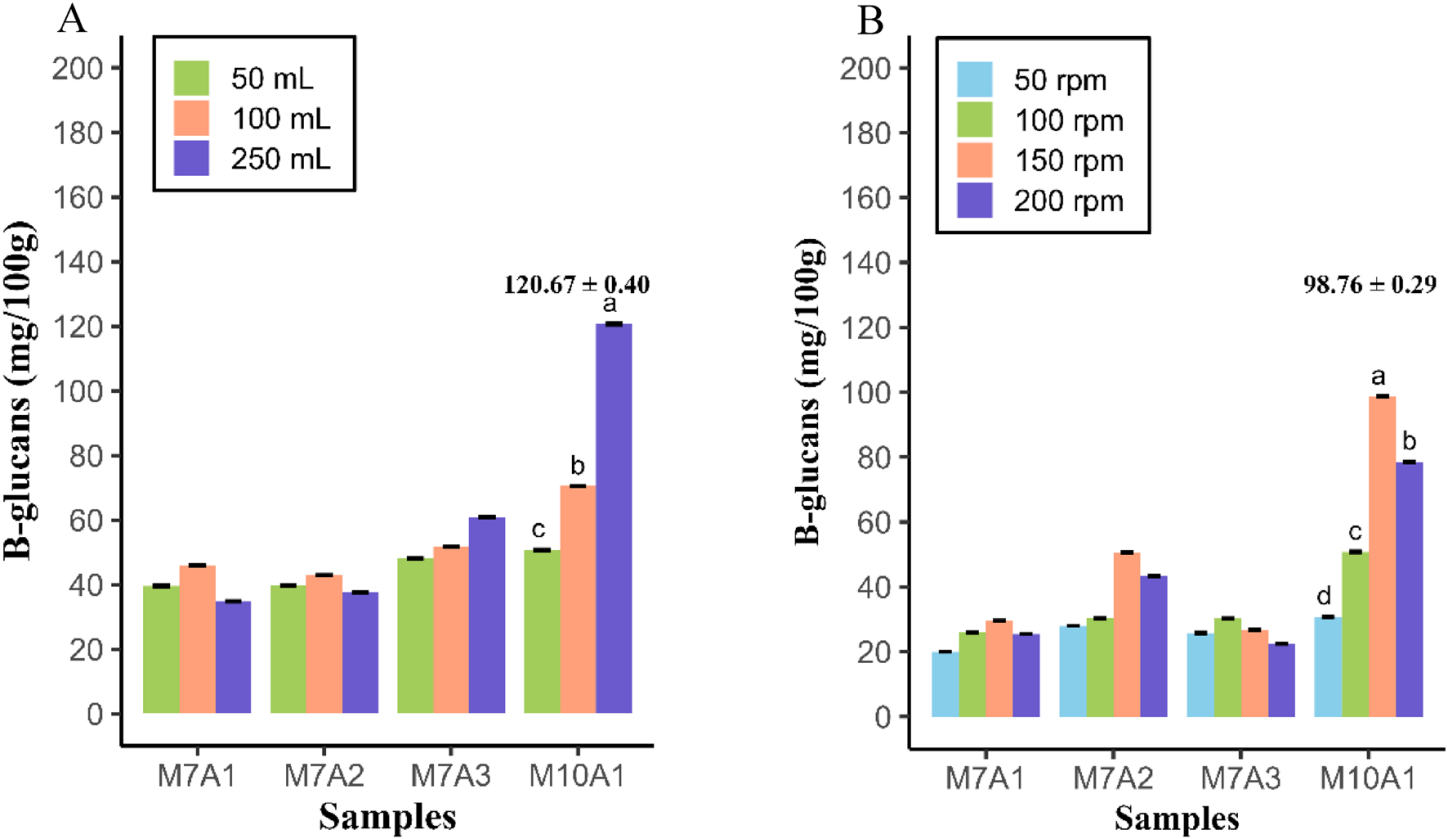
(**A**). Production of β-glucans in Erlenmeyer flasks of different volumes by the isolates M7A1, M7A2, M7A3 and M10A1. (**B**). Production of β-glucans at different stirring speeds by *Rhizopus oryzae* M7A1, M7A2, M7A3 and M10A1.

The effect of the flask volume used for fermentation and stirring speed are shown in Fig. 6A,B. The highest yield of β-glucans was produced by *Rhizopus oryzae* M10A1 at a flask volume of 250 mL (120.67 mg/100 g biomass) and at a stirring rate of 150 rpm (98.76 mg/100 g biomass). When the physical factors of fermentation were combined, *Rhizopus oryzae* M7A1 produced 276.78 mg/100 g of biomass, and M10A1 produced 397.50 mg/100 g of biomass (*p* < 0.05) (Table 4). Therefore, the parameters for producing the greatest amount of β-glucans in the liquid residues from starch processing were a six-day cultivation time, a 30 °C temperature, a pH of 6, a 250 mL volume and 150 rpm agitation.

**Table 4:** β-glucans produced by *Rhizopus oryzae* M7A1, M7A2, M7A3 and M10A1 with specified fermentation parameters.

Sample	Time of cultivation (Days)	Temperature (°C)	pH	Speed of stirring (rpm)	Volume of the flask (mL)	β-glucans (mg/100 g)
M7A1	6	35	5	150	100	276.8 ± 0.38d
M7A2	6	25	5	150	100	298.11 ± 0.20c
M7A3	6	30	4	100	250	301.08 ± 0.133b
M10A1	6	30	6	150	250	397.50 ± 0.49a

## Discussion

*Rhizopus oryzae* has a wide distribution and has been isolated from samples such as tempeh; fruits such as cucumber, guava; cereals such as oats, wheat, rice and oats; and legumes and is used in the fermentation of various types of foods that serve as a source of isolation, among others^21–25^, being ubiquitous microorganisms, the isolates of *Rhizopus oryzae* could be isolated from rice, oats and wheat.

In reference to macroscopic features found are similar to those found in MEA and PDA, which indicate that the colonies fill the plates completely in four days and that the mycelia are gray with gray to black sporangia, which gives them the characteristic color. These molds are indicated to have a cottony texture and entire margins with a growth rate of 45 mm/day at 25 °C^21,26, 27^.

The microscopic characteristics of *Rhizopus oryzae* isolates are among those reported; since the sporangiophore length is between 612 and 2337 µm, the diameter ranges from 7.0 to 20.0 µm, and the diameter ranges from 3 to 5 per group. The diameter of the spores was greater than those reported since measurements between 5.0 and 14.40 µm were obtained. These authors reported black, gray‒black and brown sporangia^21,24, 27, 28^.

A physiological characteristic used for the identification of *Rhizopus oryzae* is the growth temperature since these molds can grow at 25 °C, at 37 °C and not at 45 °C, with optimal growth temperatures between 30 and 37 °C^21,29^. This distinguishing feature allows them to be separated from other members of the genus *Rhizopus* with similar macroscopic characteristics, such as *Rhizopus stolonifer* growing at 30 °C and not at 37–45 °C, *Rhizopus microsporus* growing above 46–48 °C, *Rhizopus oligosporus* growing at a maximum temperature of 45–49 °C and *Rhizopus* sexualis growing below 30 °C^29,30^.

Based on temperature and macroscopic characteristics, the isolates M4A5, M4A10-M4A12, M8A1 and M10A3 can be classified as *Rhizopus stolonifer*, and the macroscopic characteristics are similar to those reported; the entire margins are white, brown and light gray colors depending on the ripeness of the sporangia, with soft and cottony mycelia^30–32^.

With regard to molecular identification, the results referring to the size of the band amplified using the primers V9G and LR3 are in accordance with others investigations, who reported a fragment size of approximately 1500 bp, which included the complete ITS1-5.8S-ITS2 region and the D1/D2 region of the ribosomal subunit 28S (LSU), and the consensus sequences observed for the ITS1-5.8S-ITS2 region were approximately 600 bp^26^.

Several works refer to the classification of presumptive mold isolates of *R. oryzae* that classify them at the species level into well-differentiated clades, which agrees with the classification made^21,23, 29, 33^.

In this regard, it is mentioned that molecular identification based on ITS sequences is the method of choice for the identification of organisms belonging to the *Mucoral* order, as it is a reliable method for species-level identification^34^.

Consequently, the classification carried out with macroscopic, microscopic and physiological characteristics was corroborated with molecular identification in such a way that the isolates M7A1, M7A2, M7A3 and M10A1 were identified as native isolates of *Rhizopus oryzae* from Tulcán, Ecuador.

The cell walls of molds are highly plastic and vital for cell maintenance, integrity and viability, and β-glucans are likely the most abundant and important polysaccharide, accounting for 65–90% of the total glucans^35,36^.

β-glucan biosynthesis by different types of molds is influenced by physical factors of fermentation, such as pH, temperature, oxygen concentration, agitation rate and composition of the culture medium, as they modify the physiological and metabolic behavior of molds^37,38^. These conditions are specific to each type of fungus and should therefore be investigated.

The amounts of β-glucans produced by *Rhizopus oryzae* M7A1, M7A2, M7A3 and M10A1 were less than 6.99 g/100 g when were grown in dextrose potato broth supplemented with 2 g/L of yeast extract at 25 °C in darkness and 120 rpm of agitation. These values are close to 0.9 g/100 g of biomass grown in liquid sucrose medium supplemented with yeast extract (YES) at 25 °C and 135 rpm for 7 days^39^.

The highest amount of β-glucans produced by *Rhizopus oryzae* M10A1 was produced under different temperature, stirring speed, time and culture medium conditions from those reported by the mentioned investigation. The culture media used are one of the most notable differences since the authors used nutrient media that allow adequate growth. The media used in this research were liquid residues from the extraction of potato starch, which, compared to those already mentioned, have lower amounts of nutrients.

The starch values of the culture media used are lower, and the reducing sugars are in the range between 12.50 and 62.7 g/L, 0.25 and 2.29 g/L reported respectively^40^, in liquid residues from the potato starch production process. Total solids, suspended and soluble solids, ash and nitrogen are lower than those reported in liquid residues from the wheat starch industry and in liquid residues from pea processing^10,13^.

Has been reported that these types of media are complex and can be used for the cultivation of molds since they are inexpensive and growth will depend on the species. Trace elements and sources of nitrogen and carbon are slowly metabolized. Likewise, *Rhizopus oryzae* can grow in agroindustrial waste of cassava, potato pulp, potato starch, apple and wheat residues, among others^29,41^.

By using agro-industrial residue from potato starch processing, it was possible to obtain β-glucans, which are more environmentally friendly because they can be an alternative for reducing the discharge of these wastes. In this regard, among the ethical and economic considerations to be taken into account by food processing industries is to reduce agro-industrial waste as a key factor in the sustainability of the supply chain and not to produce pollution; therefore, these wastes can be reused or revaluated by bioconversion by submerged fermentation of *R. oryzae* for the production of β-glucans, as performed in this study, with the possibility of subsequent use in industrial applications such as the development of functional foods^13,41, 42^.

## Conclusions

*Rhizopus oryzae* was isolated from cereal grains from Tulcán, Ecuador, and was identified through macroscopic, microscopic, physiological, and molecular characteristics, showing promise for nontraditional use in biomass production and isolation of β-glucans from cell walls, as this fungus has traditionally been used in Asian countries to produce fermented foods and beverages.

The isolation of fungi such as *Rhizopus oryzae* present in certain geographical regions that are adapted to specific environments is important to give them different uses from a biotechnological point of view through the production of biomass, β-glucans, and other metabolites.

The physical fermentation parameters were established for the M10A1 isolate included a fermentation time of 6 days, a temperature of 30 °C, agitation at 150 rpm, and a fermentation space volume of 250 mL. These parameters serve as a useful basis for scaling up to the bioreactor or pilot plant level.

*Rhizopus oryzae* M10A1 produced 397.50 mg/100 g of β-glucans once the physical fermentation parameters were established using potato starch processing liquid residue, which contributed to reducing the discharge of this waste to the environment and therefore less pollution in soils and bodies of water. The biotransformation of this liquid waste can be an alternative to reuse or revalorize it to perform submerged fermentation with molds such as Rhizopus oryzae; likewise, the polysaccharides obtained can be used in future industrial applications such as the development of functional foods.

Lower concentrations of β-glucans were obtained with the M7A1, M7A2, and M7A3 isolates; however, these strains can be used in the research of other fermentation processes for obtaining other components, such as enzymes, organic acids, proteins, after testing the fermentation conditions and establishing the optimal concentrations of the liquid substrate obtained from starch processing supplemented with different carbon and nitrogen sources.

## Materials and methods

### Isolation of presumptive *Rhizopus oryzae*

Wheat, rice and oat samples were purchased from 13 stores located in the city of Tulcán, Ecuador. A total of 500 g of each cereal sample was collected at random and stored in polyethylene bags at room temperature.

Molds were isolated using the direct plating method, and 10 equidistant particles were seeded onto potato dextrose agar (PDA). The plates were incubated at 25 °C for 7 days^30,43^. Once the incubation time had elapsed, those presumptive colonies that initially presented white, light gray or dark gray color and a cottony texture were selected^21^. A small proportion of the mycelium of the selected samples was placed on a fresh plate and incubated under the above conditions, and then the monosporic culture technique was applied to obtain pure cultures.

The stock culture of the pure isolates was prepared using sterile cryovial tubes of 1.5 mL with 1000 µL of 10% glycerol and preserved at − 4 °C until use. The spore suspension was prepared at a concentration of 2,0 × 10^6^ spora/mL in a 10% Tween 80 solution, and a loop was taken to be inoculated at the central point of the culture medium, as indicated in the following Sect. ^28^.

### Morphological identification of presumptive isolates of *Rhizopus oryzae*

Presumptive isolates of *Rhizopus oryzae* were cultivated on potato dextrose agar (PDA), malt extract agar (MEA) and oat agar (OA)^44^. The plates were incubated at 25, 37 and 45 °C for 4 days^21,29^. The characteristics evaluated were size, margin, colony color on the front and back of the plate, colony texture and growth temperature. Photographs of the evaluated characteristics were taken with a Nikon model 3400 camera and a stereomicroscope (BOECO, Germany).

For the microscopic observation of vegetative and reproductive structures, the microculture technique was performed using MEA, for which wet preparations were made using three drops of Shear’s solution. Observations were made using an optical microscope (Nikon Eclipse E200 MV Series, Japan) coupled with a camera (MShot, Model MS60) and the Software MShot Image Analysis System version V1.1, 2019. Measurements of sporangiophores, sporangia, sporangiospores and chlamydospores were recorded^44^.

### Molecular identification of presumptive isolates of *Rhizopus oryzae*

The isolates that were subjected to the molecular identification phase were those that had growth at 37 °C and not at 45 °C and those that presented macroscopic and microscopic characteristics typical of the microorganism being identified.

To perform molecular identification, presumptive isolates were cultured on plates of PCA at 25 °C for 4 days, and mycelia were harvested using a sterile spatula and transferred to an extraction tube to obtain genetic material. DNA was extracted using the FastDNA™ SPIN kit (MP Biomedical, U.S.A.) following the manufacturer’s instructions.

For identification, the primers V9G and LR3, which include the entire ITS1-5.8S-ITS2 region and the D1/D2 region of the subunit 28S, for sequencing the primers ITS1 and ITS4 were used^26^. The primers were synthesized by Macrogen, Corea, and the sequences were V9G: 5′-TTACGTCCCTGCCCTTTGTA-3’ and LR3: 5’-GGTCCGTGTTTCAAGAC-3’.

The PCR mixture was made in a total volume of 50 μL, which included 1.5 μL of DNA (20 ng/μL), 2.5 μL (10 μM) of each primer, 25 μL of Master Mix (FastGene® TAQ 2 × ReadyMix) and nuclease-free water to complete the reaction volume. The amplification reaction was performed on a conventional thermocycler (AllInOneCyclerTM, BiONNER, Corea) with the following cycles: an initial denaturing step of 94 °C for 5 min, followed by 35 denaturing cycles of 94 °C for 1 min, annealing to 53 °C for 1 min, elongation to 72 °C for 2 min and a final extension to 72 °C for 7 min^26^.

To visualize the product of the amplified DNA, a 1.0% agarose gel was used, which was dyed with Safe ViewTM (ABM, U.S.A.). The molecular weights of the amplified products were estimated by comparison with a molecular marker of 1 kb (DNA-Opti Plus ABM, Canada); subsequently, visualization of the amplified products was carried out in a Molecular Photodocumentary (Major Science Blue View, U.S.A.)^26^.

The PCR products were sent to a sequencing service, and consensus sequences obtained after quality control were aligned with sequences of reference strains obtained from the National Center for Biotechnology Information (NCBI), Nucleotide section Basic Local Alignment Search Tool (BLAST) (https://blast.ncbi.nlm.nih.gov/Blast.cgi, accessed on November 30, 2023) with the ClustalW tool of the MEGA-X package (64 bit for Windows). A phylogenetic tree was constructed with concatenated sequences (ITS1-ITS4) using the tool PHYLOGENY via neighbor joining with 1000 bootstrap repetitions. The sequences were deposited in GenBank with the accession numbers indicated in Fig. 3B.

## Determination of physical fermentation parameters

### Preparation of the inoculum

The inoculum was prepared using spores from the plate culture at 25 °C for 7 days in PDA. The preinoculum was prepared by adding 6 mL of sterile water to the crop, followed by manual stirring to release the spores. The spore suspension was used to inoculate the medium where submerged fermentation was performed at a concentration of 5 × 10^6^ spores/mL^13,45^.

### Conditions of submerged fermentation

Mold growth was carried out in submerged cultivation. The initial conditions used were volumetric flasks of 250 mL covered with cotton, a temperature of 30 °C, a stirring speed of 150 rpm, a pH of 5.5 and 50 mL of waste from the starch obtained previously and sterilized at 121 °C for 20 min. The cold culture medium was inoculated with the spore concentration indicated above and with the above growing conditions. The 14-day incubation time was evaluated. After determining the culture time required to obtain a higher concentration of β-glucans, we proceeded to test at temperatures of 25, 30, 35 and 40 °C and pH values of 2, 3, 4, 5, 6 and 7. The stirring speeds used were 50, 100, 150 and 200 rpm, and the Erlenmeyer volumes used were 50, 100 and 250 mL^13^.

The flasks were constantly agitated in an orbital agitator (BOECO ES-20/80, Germany), and for cultivation time, biomass samples were taken every 24 h. In the other conditions, the samples were taken at the set time by the culture time test as it was where the highest amount of β-glucans was produced. The biomass was washed with deionized water repeatedly until it was free of the culture broth, subsequently dried at 65 °C for 24 h, allowed to cool at room temperature and weighed until later use in the extraction process of polysaccharides from the cell walls^46^.

The one-variable-at-a-time (OVAT) approach was used to evaluate the effect of each physical parameter separately^47^. Once the crop parameters were established for each of the *Rhizopus oryzae* isolates, submerged culture was performed to determine the number of β-glucans produced by the combination of previously established conditions.

### Determination of the chemical composition of the culture medium

Total solids, suspended solids and soluble solids were determined (Method No 920.151), total nitrogen (No 2001.11), total protein (%N × 6.25), ash (No 920.153), total starch (Method No 996.11) reducing and total sugars (Method No 32024-32-025)^48^.

### Extraction of polysaccharides from cell walls

The extraction of polysaccharides from the cell walls was carried out, the dried mycelium was disintegrated in a rotor mill (Tecnal de rotor R-TE-651/2, Brazil). The resulting material was immersed in 95% ethanol and refluxed for 3 h to degrease the sample in a Soxhlet apparatus. The degreased residue was dried and extracted with a water ratio of 1:40 (w/w) at a temperature of 121 °C for 1 h and then filtered through a 200-mesh nylon sieve. The extraction was repeated two more times. The total filtrate was centrifuged at 2000 × g for 15 min (HERMLE Z206A, Germany), and the supernatant was concentrated to 1/5 of the initial volume and precipitated with 80% ethanol at 4 °C for 24 h. The filtrate was then centrifuged at the above mentioned speed, collected and purified. Subsequently, the sample was centrifuged at the speed indicated above, collected and purified^8,49^.

### Purification of β-glucans from cell walls

The purification of β-glucans was performed by washing the obtained precipitates with ethanol followed by acetone and ether (1:3 V/V) in successive order, deproteinizing them with CHCl3-n-BuOH (chloroform n-butanol) at a 5:1 ratio (v/v) 5 times, dialyzing them with a 12–14 kDa membrane against distilled water for 72 h and finally lyophilized^8,23^. The amount of β-glucans was calculated by determining the total sugars via the phenol‒sulfuric method^49^.

Analyses were performed in triplicate, and the results are presented as the mean ± standard deviation. Microscopic measurements are the average of 30 measurements. Macroscopic measurements are the average of 9 measurements. They were compared by ANOVA analysis and the least significant difference (LSD) test with a confidence interval of 95%. Statistical analyses were performed and graphs were generated using the free software RStudio Version 2023.06.2 + 561.

### Supplementary Information

Supplementary Information.

## Data Availability

Data from the sequences of *Rhizopus oryzae* M7A1, M7A2, M7A3 and M10A1 were deposited in the NCBI and can be found at the following links: https://www.ncbi.nlm.nih.gov/nuccore/OR775417 https://www.ncbi.nlm.nih.gov/nuccore/OR775423 https://www.ncbi.nlm.nih.gov/nuccore/OR775418 https://www.ncbi.nlm.nih.gov/nuccore/OR775424 https://www.ncbi.nlm.nih.gov/nuccore/OR775419 https://www.ncbi.nlm.nih.gov/nuccore/OR775426 https://www.ncbi.nlm.nih.gov/nuccore/OR775420 https://www.ncbi.nlm.nih.gov/nuccore/OR775425
