# Supplementary material for: Polyphasic identification of Rhizopus oryzae and evaluation of physical fermentation parameters in potato starch processing liquid waste for β-glucan production
Source: Sci Rep. 2024 Jun 28;14:14913. doi: 10.1038/s41598-024-66000-5 (PMC11213850; doi:10.1038/s41598-024-66000-5)
Supplement: Supplementary file 1 — Supplementary Information. [file 41598_2024_66000_MOESM1_ESM.docx]

**β-glucans of cell walls: Identification of *Rhizopus oryzae* and determination of physical fermentation parameters in potato starch processing liquid waste**

**Miguel Anchundia^1,2*^, Gualberto León-Revelo^1^, Stalin Santacruz^3^ and Freddy Torres^1^**

^1^ School of Food Engineering, Universidad Politécnica Estatal del Carchi, 040101 Tulcán, Ecuador

^2^ Faculty of Sciences, Universidad de la República, 11200 Montevideo, Uruguay.

^3^ School of Agroindustrial Engineering, Universidad Laica Eloy Alfaro de Manabí, 130222 Manta, Ecuador

. *Corresponding author: miguel.anchundia@upec.edu.ec


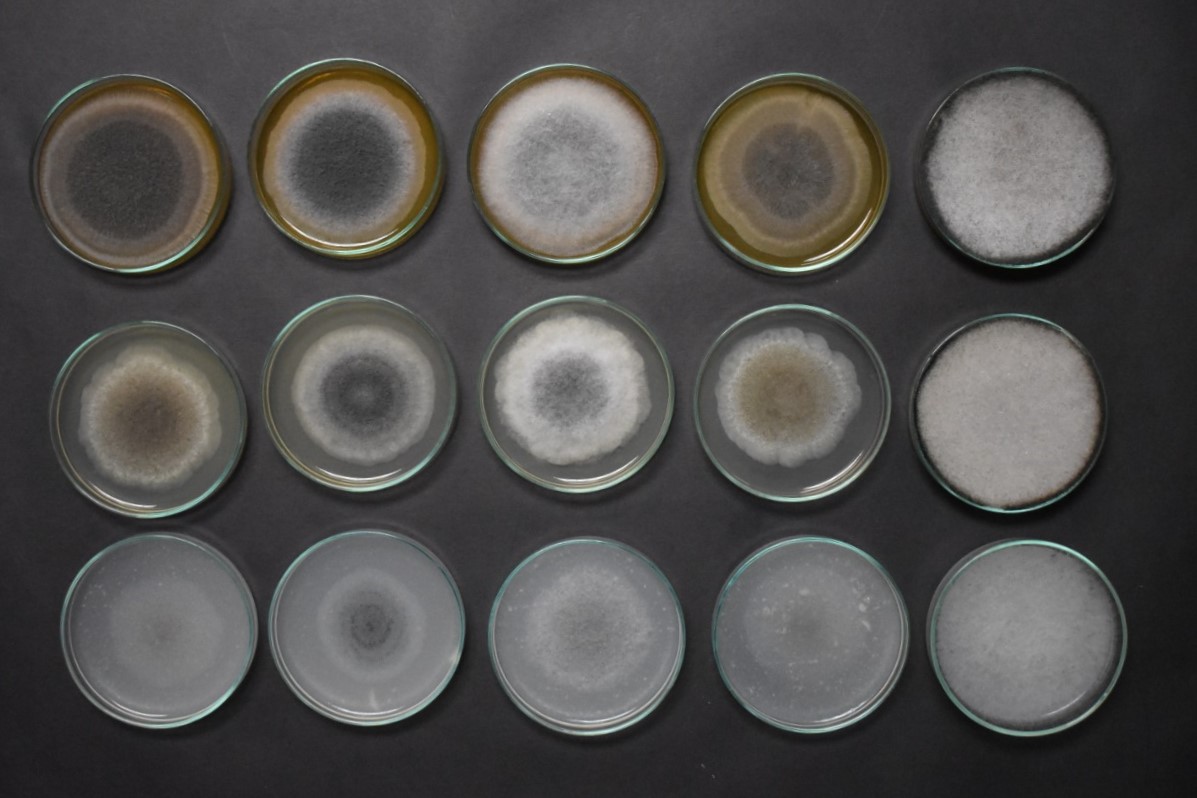


**Supplementary Figure S1.** Colonies of presumptive *Rhizopus oryzae* grown at 25 °C for 4 days. Column 1= M4A1, column 2 = M4A2, column 3 = M4A3, column 4 = M4A4 to column 5 = M4A5. Row 1 shows the MEA isolates, row 2 shows the PDA isolates, and row 3 shows the OA isolates.


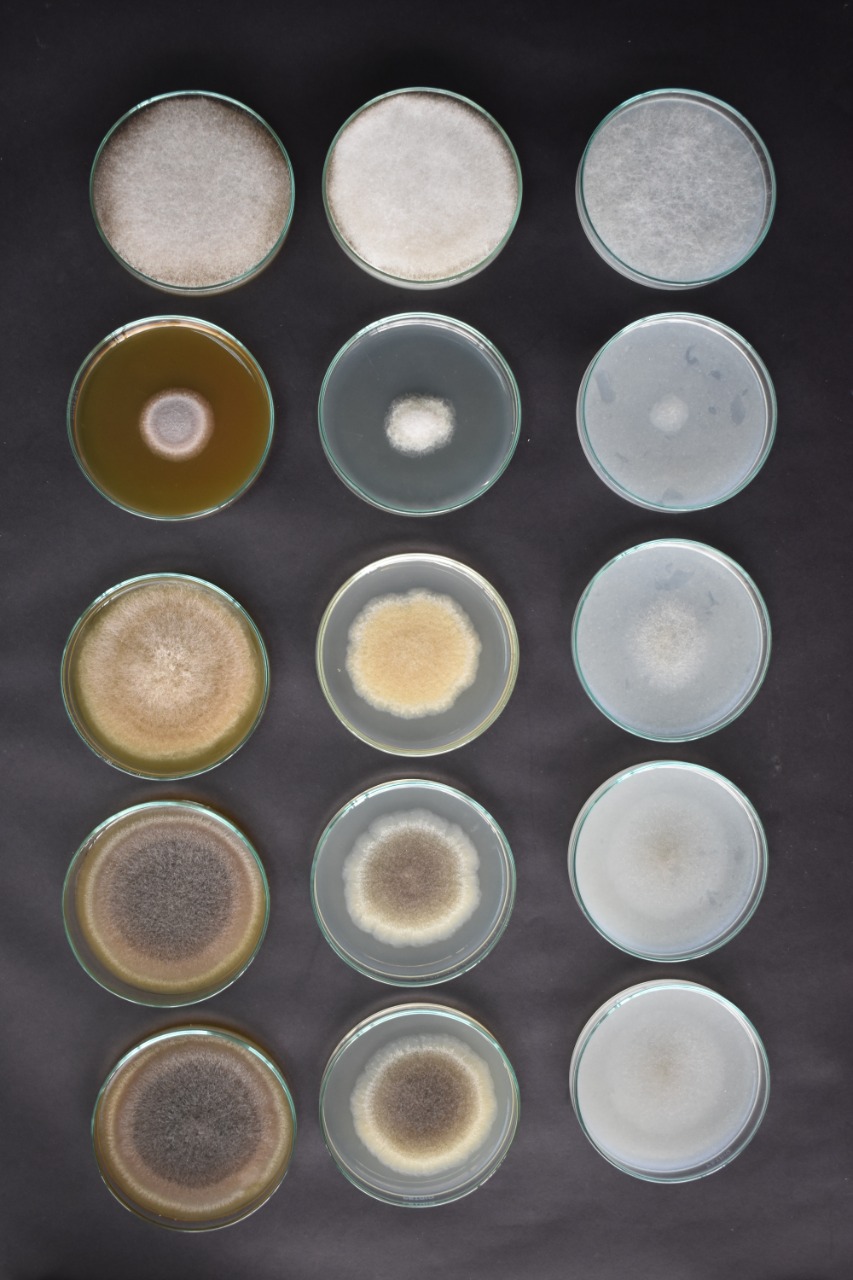


**Supplementary Figure S2**. Colonies of presumptive *Rhizopus oryzae* grown at 25 °C for 4 days. Column 1 = M4A6, column 2 = M4A7, column 3 = M4A8, column 4 = M4A9 to column 5 = M4A10. Row 1 shows isolates on MEA, row 2 shows isolates on PDA, and row 3 shows isolates on OA.

**Supplementary Table S1**. Macroscopic characteristics of M4A2 and M4A9 isolates on MEA, PDA, and OA grown at 25 °C, 37 °C, and 45 °C for 4 days.

| **Sample** | **Agar** | **Temperature (⁰C)** | **Surface color** | **Back color** | **Colony size (cm)^1^** | **Margin** | **Growth rate** | **Texture** |  |
| --- | --- | --- | --- | --- | --- | --- | --- | --- | --- |
|  |  |  |  |  |  |  |  |  |  |
| M4A2 Oatmeal | MEA | 25 | Dark gray in the center, white in the edge | Cream | 7.60 ± 0.26 | Entire | Fast | Cottony |  |
|  |  | 37 | Dark gray | Cream | 9.00 ± 0.00 | Entire | Fast | Cottony |  |
|  |  | 45 | Brown | Cream | 7.40 ± 0.10 | Entire | Fast | Cottony |  |
|  | PDA | 25 | Dark gray in the center, white in the edge | White | 7.90 ± 0.26 | Entire | Medium | Cottony |  |
|  |  | 37 | Dark gray | White | 9.40 ± 0.00 | Entire | Fast | Cottony |  |
|  |  | 45 | Dark brown | Red | 8.83 ± 0.29 | wavy | Fast | Cottony |  |
|  | OA | 25 | Dark gray in the center, white in the edge | White | 4.27 ± 0.25 | Entire | Medium | Cottony |  |
|  |  | 37 | Dark gray in the center, white in the edge | White | 9.00 ± 0.00 | Entire | Fast | Cottony |  |
|  |  | 45 | Brown | Cream | 9.00 ± 0.00 | Entire | Fast | Cottony |  |
| M4A9 Oatmeal | MEA | 25 | Light gray in the center, White in the edge | Orange | 3.31 ± 0.31 | Entire | Slow | Cottony |  |
|  |  | 37 | Light gray | Orange | 9.00 ± 0.00 | Entire | Fast | Cottony |  |
|  |  | 45 | White | Cream | 3.40 ± 0.21 | Entire | Slow | Cottony |  |
|  | PDA | 25 | White | White | 3.63 ± 0.14 | wavy | Slow | Cottony |  |
|  |  | 37 | White | White | 9.00 ± 0.00 | Entire | Fast | Cottony |  |
|  |  | 45 | White | White | 3.13 ± 0.31 | wavy | Slow | Cottony |  |
|  | OA | 25 | White | White | 2.20 ± 0.00 | wavy | Slow | Cottony |  |
|  |  | 37 | White | White | 8.50 ± 0.00 | Entire | Fast | Cottony |  |
|  |  | 45 | White | White | 2.00 ± 0.00 | Entire | Slow | Cottony |  |


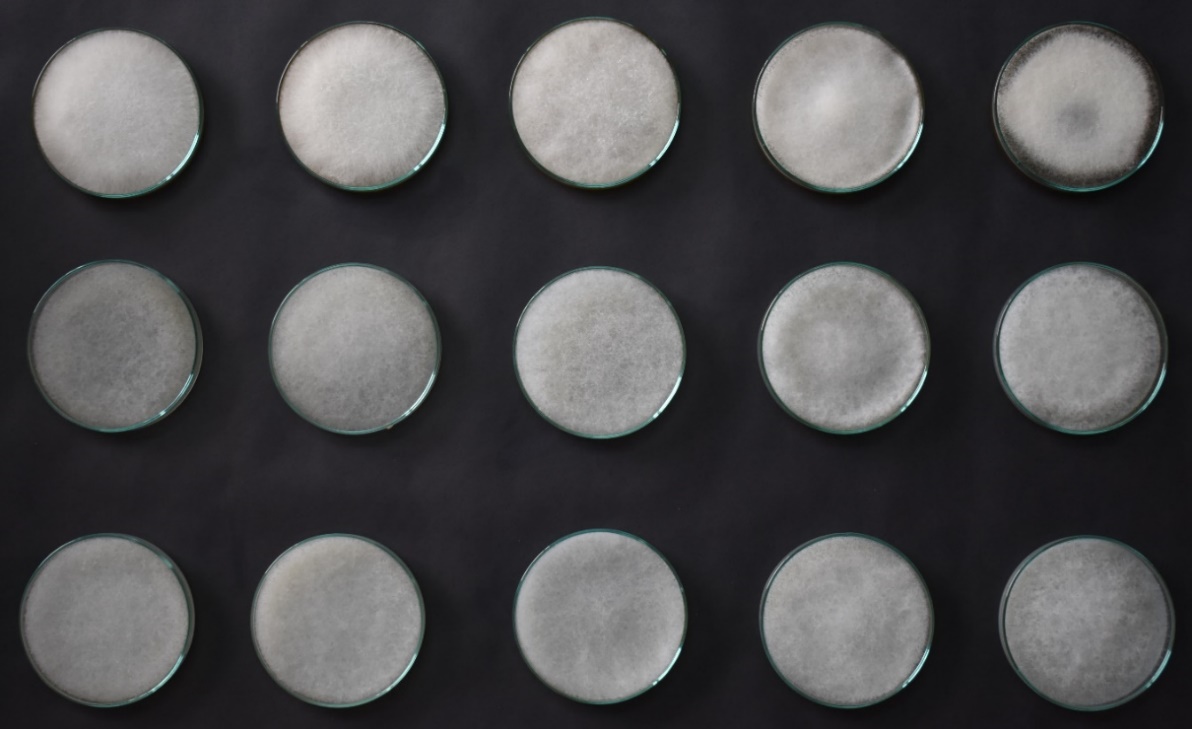


**Supplementary Figure S3.** Colonies of presumptive isolates of *Rhizopus oryzae* grown at 25 °C for 4 days. Column 1 = M4A11, column 2 = M4A12, column 3 = M7A1, column 4 = M7A2 to column 5 = M7A3. Row 1 shows the MEA isolates, row 2 shows the PDA isolates, and row 3 shows the OA isolates.

**Supplementary Table S2**. Macroscopic characteristics of M4A1, M4A5-M4A8, M4A10-M4A12, M8A1 and M10A3 isolates on MEA, PDA, and OA grown at 25 °C, 37 °C, and 45 °C for 4 days.

| **Sample** | **Agar** | **Temperature (⁰C)** | **Surface color** | **Back color** | **Colony size (cm)^1^** | **Margin** | **Growth rate** | **Texture** |  |
| --- | --- | --- | --- | --- | --- | --- | --- | --- | --- |
|  |  |  |  |  |  |  |  |  |  |
| M4A1 Rice | MEA | 25 | Dark gray in the center, white in the edge | Cream | 8.83 ± 0.29 | Entire | Fast | Cottony |  |
|  |  | 37 | There was no growth | | | | | |  |
|  |  | 45 | There was no growth | | | | | |  |
|  | PDA | 25 | Dark gray in the center, white in the edge | White | 7.00 ± 0.20 | Entire | Fast | Cottony |  |
|  |  | 37 | There was no growth | | | | | |  |
|  |  | 45 | There was no growth | | | | | |  |
|  | OA | 25 | Dark gray in the center, white in the edge | White | 4.40 ± 0.10 | Entire | Medium | Cottony |  |
|  |  | 37 | There was no growth | | | | | |  |
|  |  | 45 | There was no growth | | | | | |  |
| M4A5 Oatmeal | MEA | 25 | White in the center, dark gray in the edge | Cream | 9.00 ± 0.00 | Entire | Fast | Cottony |  |
|  |  | 37 | There was no growth | | | | | |  |
|  |  | 45 | There was no growth | | | | | |  |
|  | PDA | 25 | White in the center, gray dark in the edge | Cream | 9.00 ± 0.00 | Entire | Fast | Cottony |  |
|  |  | 37 | There was no growth | | | | | |  |
|  |  | 45 | There was no growth | | | | | |  |
|  | OA | 25 | White | Cream | 9.00 ± 0.00 | Entire | Fast | Cottony |  |
|  |  | 37 | There was no growth | | | | | |  |
|  |  | 45 | There was no growth | | | | | |  |
| M4A6 Oatmeal | MEA | 25 | Light gray in the center, white in the edge | Dark brown | 8.00 ± 0.00 | Entire | Fast | Cottony |  |
|  |  | 37 | There was no growth | | | | | |  |
|  |  | 45 | There was no growth | | | | | |  |
|  | PDA | 25 | Dark gray in the center, white in the edge | White | 6.73 ± 0.15 | Entire | Medium | Cottony |  |
|  |  | 37 | There was no growth | | | | | |  |
|  |  | 45 | There was no growth | | | | | |  |
|  | OA | 25 | Light gray in the center and the edge | White | 5.83 ± 0.47 | Entire | Medium | Cottony |  |
|  |  | 37 | There was no growth | | | | | |  |
|  |  | 45 | There was no growth | | | | | |  |
| M4A7 Oatmeal | MEA | 25 | Light gray in the center, white in the edge | Dark brown | 9.00 ± 0.00 | Entire | Fast | Cottony |  |
|  |  | 37 | There was no growth | | | | | |  |
|  |  | 45 | There was no growth | | | | | |  |
|  | PDA | 25 | Dark gray in the center, white in the edge | White | 6.63 ± 0.12 | Entire | Medium | Cottony |  |
|  |  | 37 | There was no growth | | | | | |  |
|  |  | 45 | There was no growth | | | | | |  |
|  | OA | 25 | Dark gray in the center, white in the edge | White | 5.70 ± 0.00 | Entire | Medium | Cottony |  |
|  |  | 37 | There was no growth | | | | | |  |
|  |  | 45 | There was no growth | | | | | |  |
| M4A8 Oatmeal | MEA | 25 | Light gray | Orange | 8.95 ± 0.21 | Entire | Fast | Cottony |  |
|  |  | 37 | There was no growth | | | | | |  |
|  |  | 45 | There was no growth | | | | | |  |
|  | PDA | 25 | Light brown in the center, White in the edge | White | 7.40 ± 0.10 | Entire | Medium | Cottony |  |
|  |  | 37 | There was no growth | | | | | |  |
|  |  | 45 | There was no growth | | | | | |  |
|  | OA | 25 | Light gray in the center, white in the edge | White | 5.00 ± 0.00 | Entire | Medium | Cottony |  |
|  |  | 37 | There was no growth | | | | | |  |
|  |  | 45 | There was no growth | | | | | |  |
| M4A10 Oatmeal | MEA | 25 | Light gray in the center, dark gray in the edge | Cream | 9.00 ± 0.00 | Entire | Fast | Cottony |  |
|  |  | 37 | There was no growth | | | | | |  |
|  |  | 45 | There was no growth | | | | | |  |
|  | PDA | 25 | Light gray in the center, dark gray in the edge | White | 9.00 ± 0.00 | Entire | Fast | Cottony |  |
|  |  | 37 | There was no growth | | | | | |  |
|  |  | 45 | There was no growth | | | | | |  |
|  | OA | 25 | Light gray | White | 9.00 ± 0.00 | Entire | Fast | Cottony |  |
|  |  | 37 | There was no growth | | | | | |  |
|  |  | 45 | There was no growth | | | | | |  |
| M4A11 Oatmeal | MEA | 25 | Light gray in the center, dark gray in the edge | Orange | 9.00 ± 0.00 | Entire | Fast | Cottony |  |
|  |  | 37 | There was no growth | | | | | |  |
|  |  | 45 | There was no growth | | | | | |  |
|  | PDA | 25 | White in the center, light gray in the edge | White | 9.00 ± 0,00 | Entire | Fast | Cottony |  |
|  |  | 37 | There was no growth | | | | | |  |
|  |  | 45 | There was no growth | | | | | |  |
|  | OA | 25 | White | White | 9.00 ± 0.00 | Entire | Fast | Cottony |  |
|  |  | 37 | There was no growth | | | | | |  |
|  |  | 45 | There was no growth | | | | | |  |
| M4A12 Rice | MEA | 25 | Light gray in the center, dark gray in the edge | Orange | 9.00 ± 0.00 | Entire | Fast | Cottony |  |
|  |  | 37 | There was no growth | | | | | |  |
|  |  | 45 | There was no growth | | | | | |  |
|  | PDA | 25 | White in the center, light gray in the edge | White | 9.00 ± 0.00 | Entire | Fast | Cottony |  |
|  |  | 37 | There was no growth | | | | | |  |
|  |  | 45 | There was no growth | | | | | |  |
|  | OA | 25 | White | White | 9.00 ± 0.00 | Entire | Fast | Cottony |  |
|  |  | 37 | There was no growth | | | | | |  |
|  |  | 45 | There was no growth | | | | | |  |
| M8A1  Rice | MEA | 25 | Light gray in the center, black in the edge | Orange | 9.00 ± 0.00 | Entire | Fast | Cottony |  |
|  |  | 37 | There was no growth | | | | | |  |
|  |  | 45 | There was no growth | | | | | |  |
|  | PDA | 25 | Light gray in the center, black in the edge | Cream | 9.00 ± 0.00 | Entire | Fast | Cottony |  |
|  |  | 37 | There was no growth | | | | | |  |
|  |  | 45 | There was no growth | | | | | |  |
|  | OA | 25 | White in the center, dark gray in the edge | White | 9.00 ± 0.00 | Entire | Fast | Cottony |  |
|  |  | 37 | There was no growth | | | | | |  |
|  |  | 45 | There was no growth | | | | | |  |
| M10A3  Rice | MEA | 25 | Light gray in the center, black in the edge | Orange | 5.50 ± 0.30 | wavy | Medium | Cottony |  |
|  |  | 37 | There was no growth | | | | | |  |
|  |  | 45 | There was no growth | | | | | |  |
|  | PDA | 25 | White in the center, black in the edge | White | 9.00 ± 0.00 | Entire | Fast | Cottony |  |
|  |  | 37 | There was no growth | | | | | |  |
|  |  | 45 | There was no growth | | | | | |  |
|  | OA | 25 | Light gray | White | 9.00 ± 0.00 | Entire | Fast | Cottony |  |
|  |  | 37 | There was no growth | | | | | |  |
|  |  | 45 | There was no growth | | | | | |  |


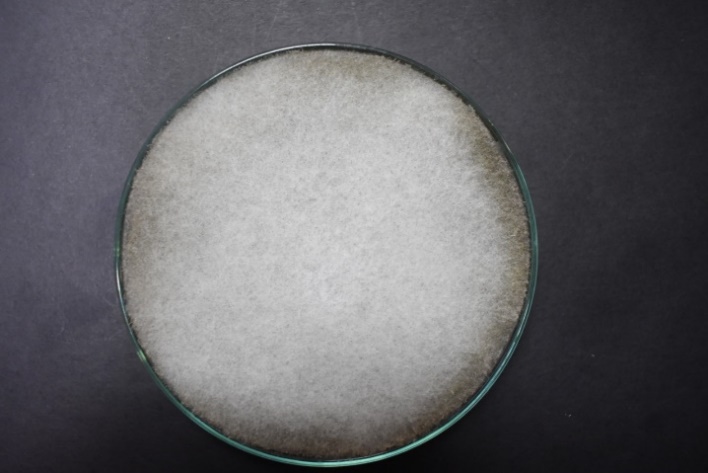


AD


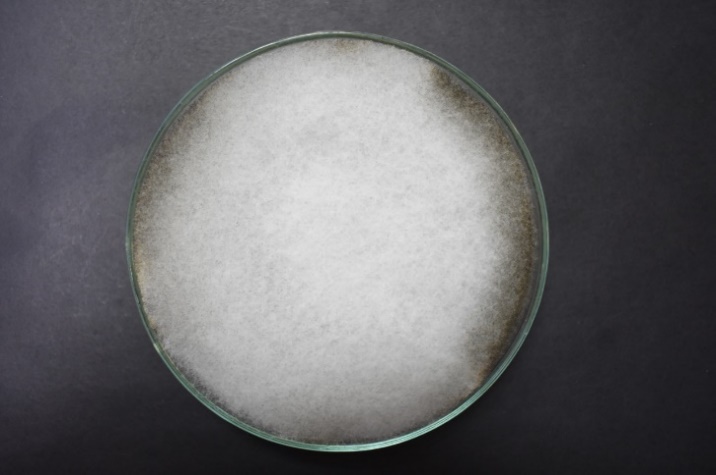


BD


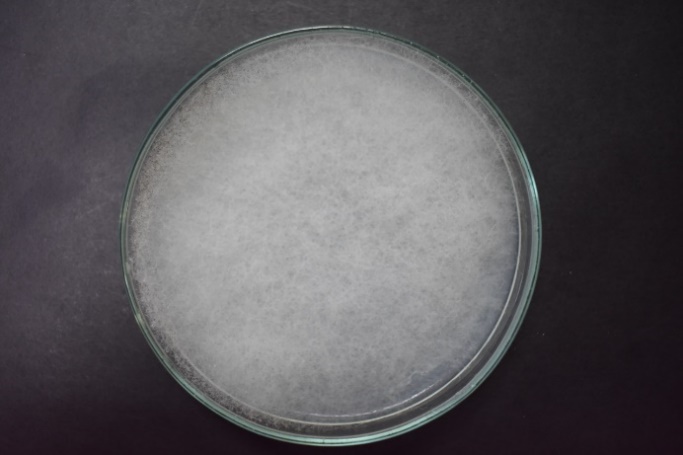


CD


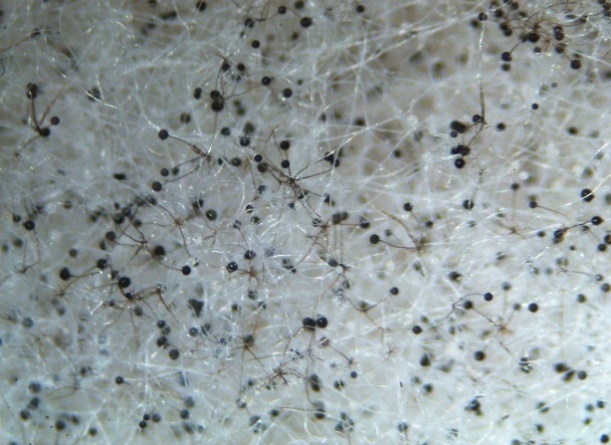


DD


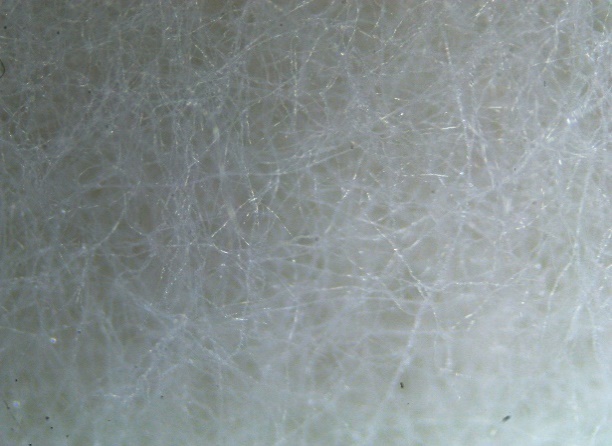


ED


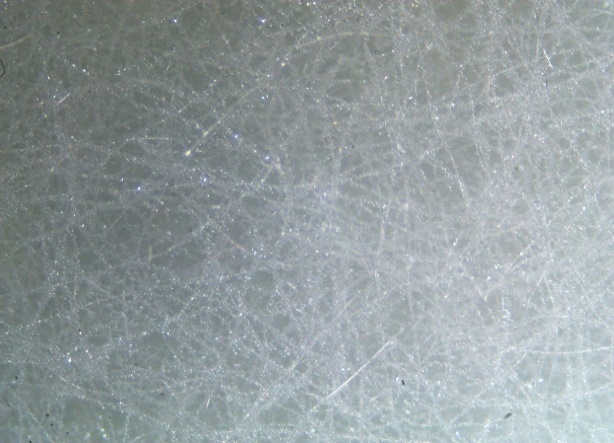


FD


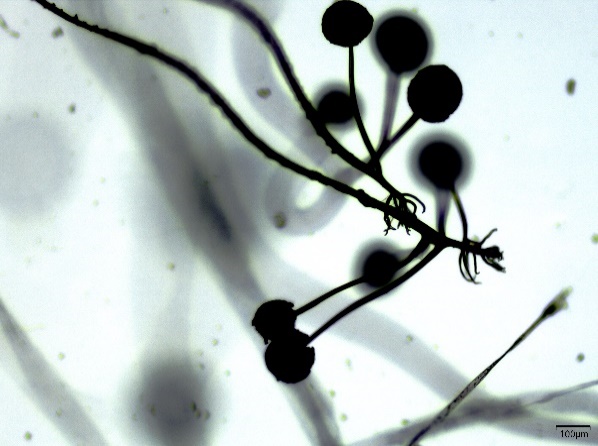


GD


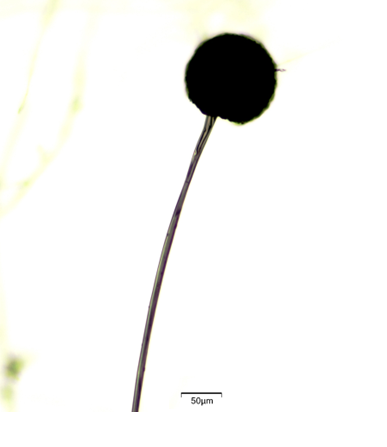


HD


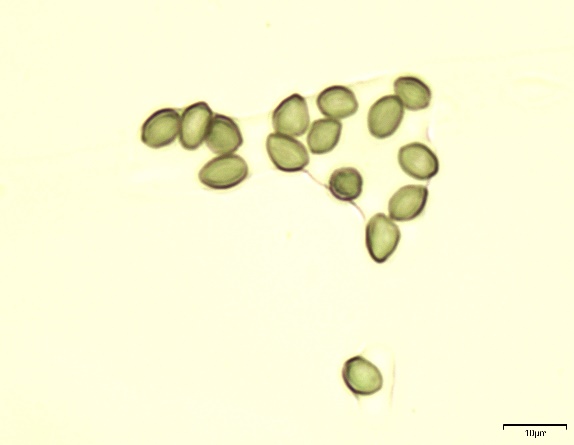


ID

**Supplementary Figure S4.** Presumptive *Rhizopus oryzae* colonies of the M7A1 isolate. A-C. Colonies were grown on MEA, PDA and OA at 37 °C for 4 days. D-E. Texture of the colonies grown in MEA, PDA and OA at 25 °C for 4 days. F-H. Sporangiophores and sporangiospores were grown on MEA agar at 25 °C. G bar, 100 µm. H bar, 50 µm. I bar, 10 µm.


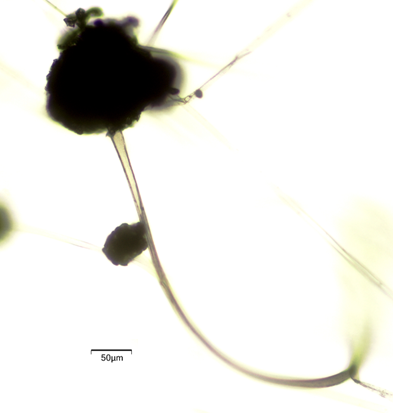


GD


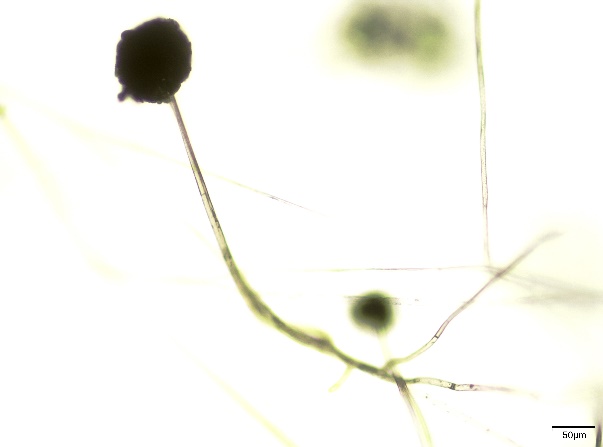


HD


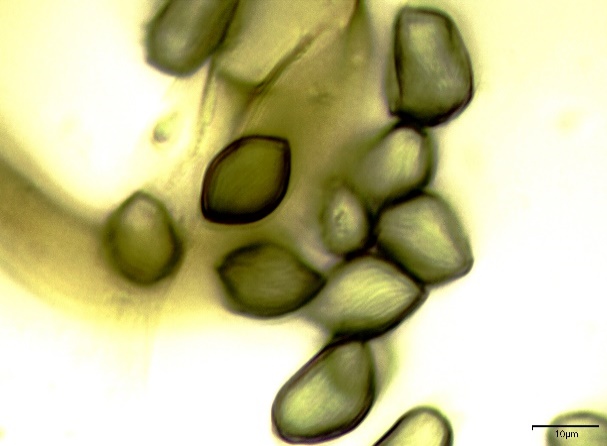


ID


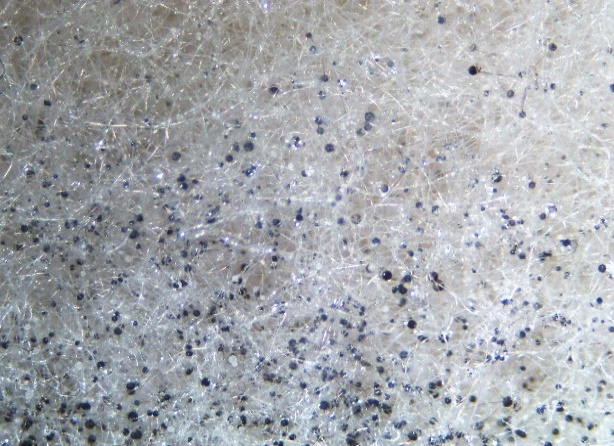


DD


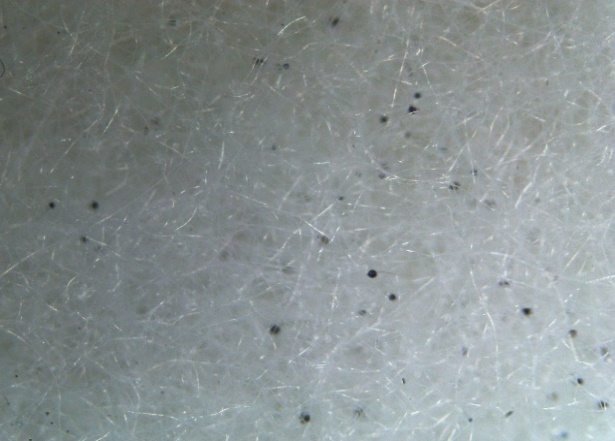


ED


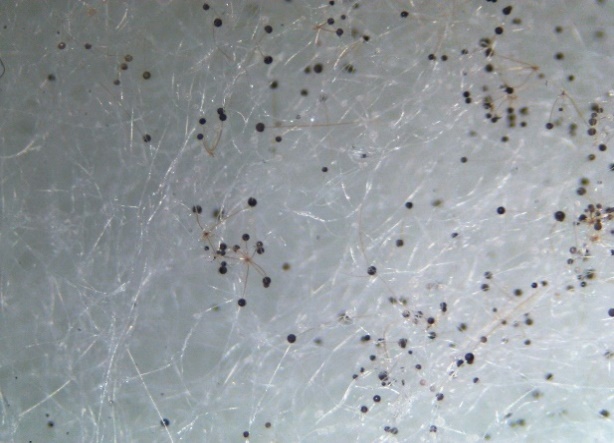


FD


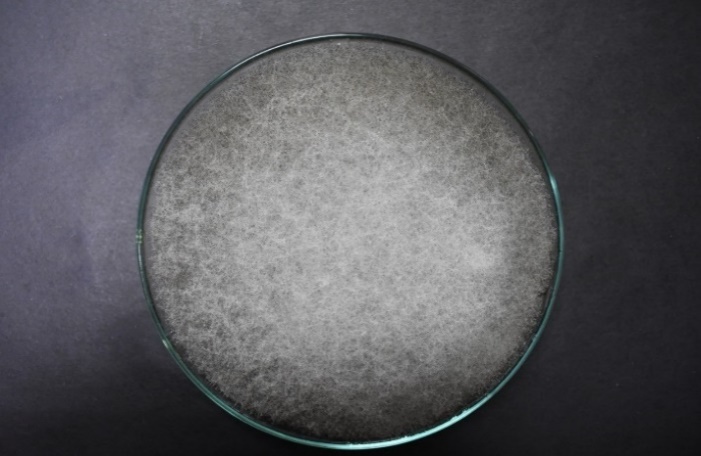


AD


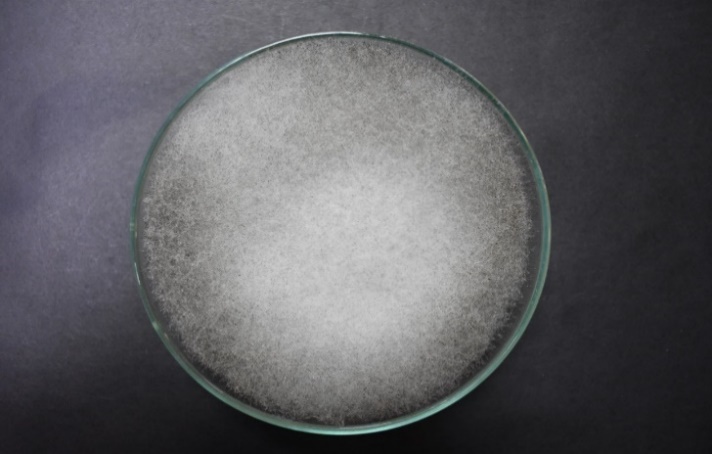


BD


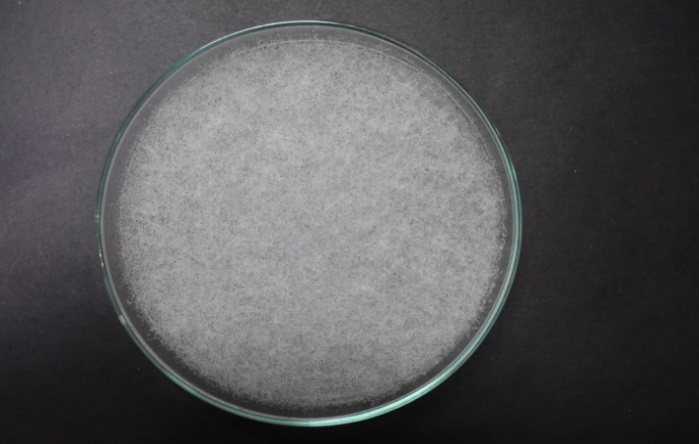


CD

**Supplementary Figure S5.** Presumptiv*e Rhizopus oryzae* colonies of the M7A2 isolate. A-C. Colonies were grown on MEA, PDA and OA at 37 °C for 4 days. D-E. Texture of the colonies grown on MEA, PDA and OA at 25 °C for 4 days. F-H. Sporangiophores and sporangiospores were grown on MEA agar at 25 °C. G bar, 50 µm. H bar, 50 µm. I bar, 10 µm.


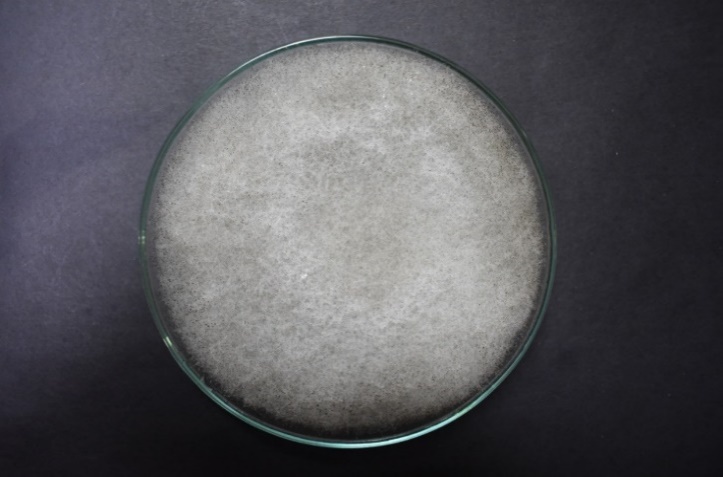


BD


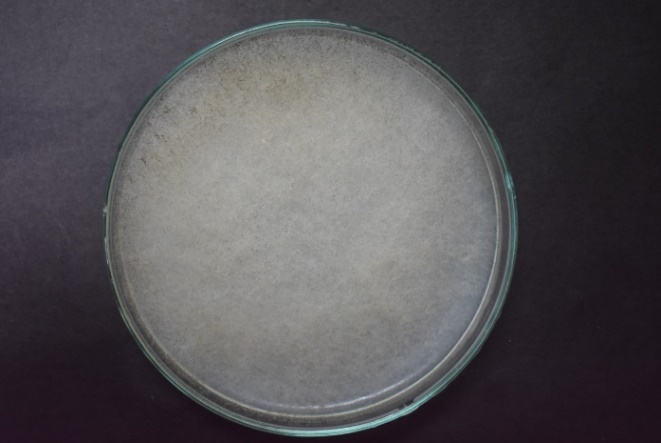


CD


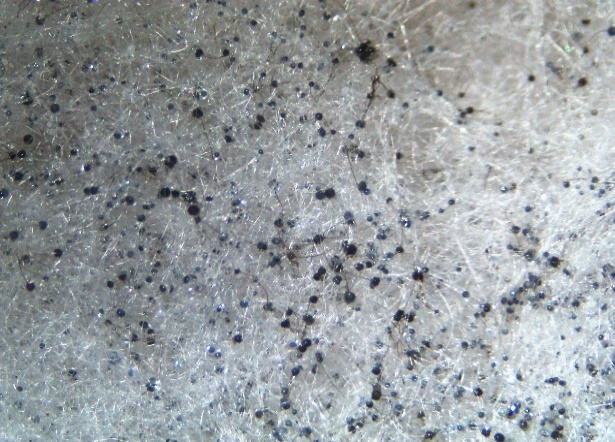


D


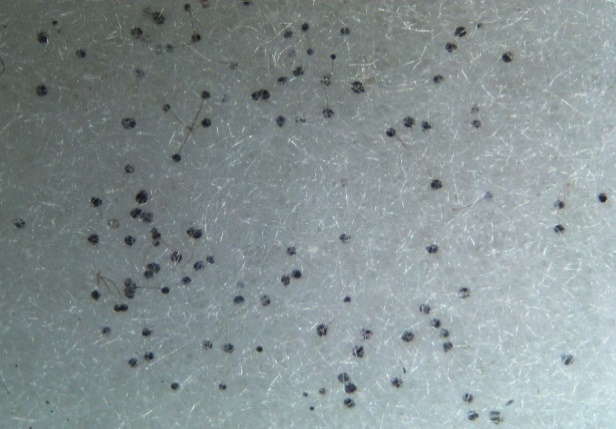


ED


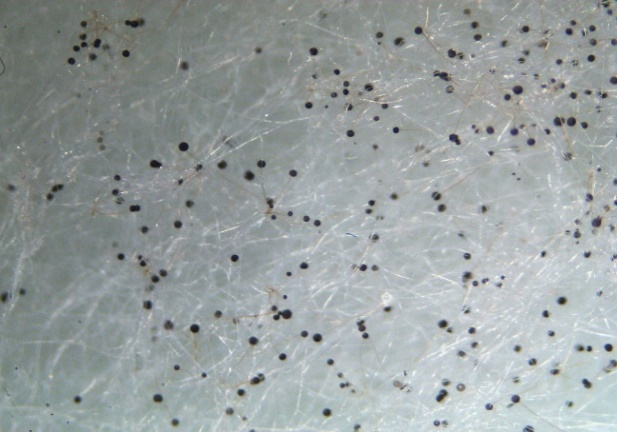


FD


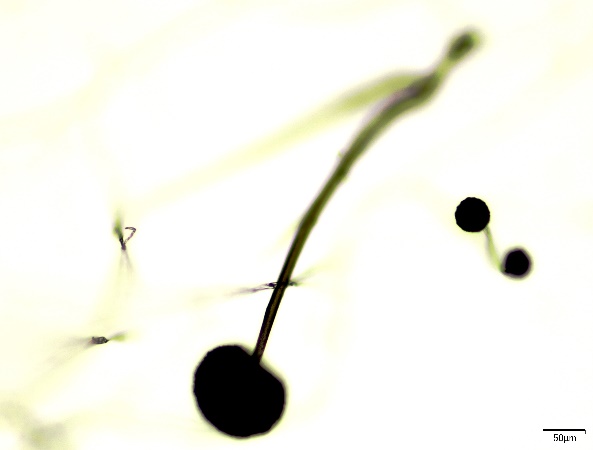


GD


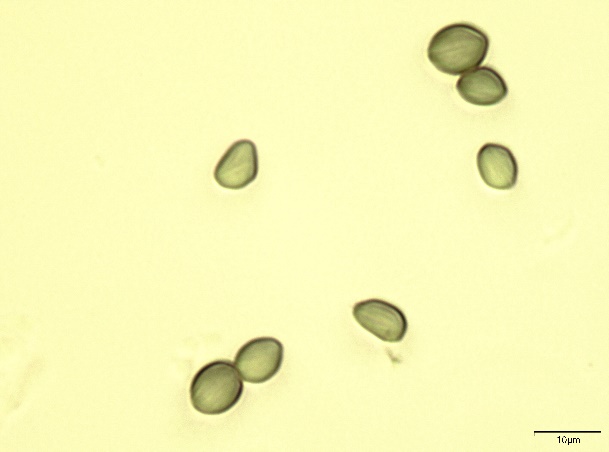


ID


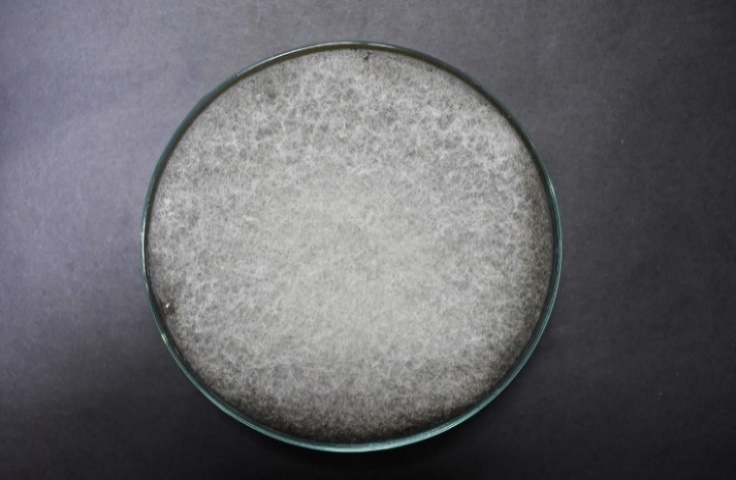


A


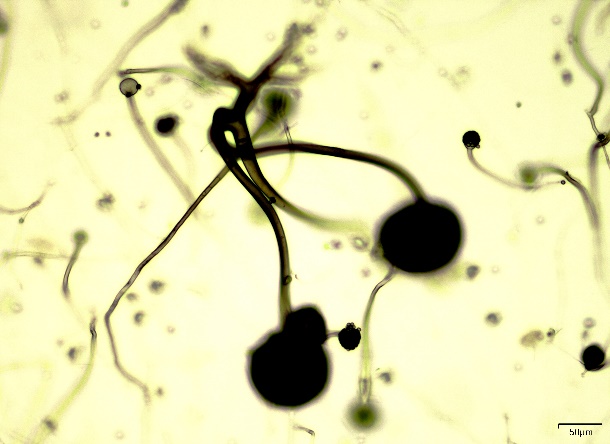


HD

**Supplementary Figure S6.** Presumptive *Rhizopus oryzae* colonies of the M7A3 isolate. A-C. Colonies were grown on MEA, PDA and OA at 37 °C for 4 days. D-E. Texture of the colonies grown on MEA, PDA and OA at 25 °C for 4 days. F-H. Sporangiophores and sporangiospores were grown on MEA at 25 °C. G bar, 50 µm. H bar, 50 µm. I bar, 10 µm.

**Supplementary Table S3**. Macroscopic characteristics of M7A1 and M7A2 isolates on MEA, PDA, and OA grown at 25 °C, 37 °C, and 45 °C for 4 days.

| **Sample** | **Agar** | **Temperature (⁰C)** | **Surface color** | **Back color** | **Colony size (cm)^1^** | **Margin** | **Growth rate** | **Texture** |
| --- | --- | --- | --- | --- | --- | --- | --- | --- |
| M7A1 Wheat | MEA | 25 | Light gray in the center, dark gray in the edge | Orange | 9.00 ± 0.00 | Entire | Fast | Cottony |
|  |  | 37 | Dark gray in the center, black in the edge | Orange | 9.00 ± 0.00 | Entire | Fast | Cottony |
|  |  | 45 | There was no growth | | | | | |
|  | PDA | 25 | White in the center, light gray in the edge | White | 9.00 ± 0,00 | Entire | Fast | Cottony |
|  |  | 37 | Dark gray in the center, black in the edge | White | 9.00 ± 0.00 | Entire | Fast | Cottony |
|  |  | 45 | There was no growth | | | | | |
|  | OA | 25 | White | White | 9.00 ± 0.00 | Entire | Fast | Cottony |
|  |  | 37 | Light gray | White | 9.00 ± 0.00 | Entire | Fast | Cottony |
|  |  | 45 | There was no growth | | | | | |
| M7A2 Rice | MEA | 25 | Light gray in the center, dark gray in the edge | Orange | 9.00 ± 0.00 | Entire | Fast | Cottony |
|  |  | 37 | Dark gray in the center, black in the edge | Orange | 9.00 ± 0.00 | Entire | Fast | Cottony |
|  |  | 45 | There was no growth | | | | | |
|  | PDA | 25 | White | White | 9.00 ± 0.00 | Entire | Fast | Cottony |
|  |  | 37 | White in the center, light gray in the edge | White | 9.00 ± 0.00 | Entire | Fast | Cottony |
|  |  | 45 | There was no growth | | | | | |
|  | OA | 25 | White | White | 9.00 ± 0.00 | Entire | Fast | Cottony |
|  |  | 37 | White | White | 9.00 ± 0.00 | Entire | Fast | Cottony |
|  |  | 45 | There was no growth | | | | | |

**^1^ Average of 9 colonies ± standard deviation**

**Supplementary Table S4.** Macroscopic characteristics of M7A3 and M10A1 isolates cultivated on MEA, PDA and OA at 25 °C, 37 °C and 45 °C for 4 days.

| **Sample** | **Agar** | **Temperature (⁰C)** | **Surface color** | **Back color** | **Colony size (cm)^1^** | **Margin** | **Growth rate** | **Texture** |
| --- | --- | --- | --- | --- | --- | --- | --- | --- |
|  |  |  |  |  |  |  |  |  |
| M7A3 Wheat | MEA | 25 | Light gray | Cream | 9.00 ± 0.00 | Entire | Fast | Cottony |
|  |  | 37 | Light gray | Cream | 9.00 ± 0.00 | Entire | Fast | Cottony |
|  |  | 45 | There was no growth | | | | | |
|  | PDA | 25 | White | White | 9.00 ± 0.00 | Entire | Fast | Cottony |
|  |  | 37 | Light gray | White | 9.00 ± 0.00 | Entire | Fast | Cottony |
|  |  | 45 | There was no growth | | | | | |
|  | OA | 25 | White | White | 9.00 ± 0.00 | Entire | Fast | Cottony |
|  |  | 37 | White | White | 9.00 ± 0.00 | Entire | Fast | Cottony |
|  |  | 45 | There was no growth | | | | | |
|  |  | 45 | There was no growth | | | | | |

**Supplementary Table S5.** Macroscopic characteristics of M4A3 and M4A4 isolates on MEA, PDA, and OA grown at 25 °C, 37 °C, and 45 °C for 4 days.

| **Sample** | **Agar** | **Temperature (⁰C)** | **Surface color** | **Back color** | **Colony size (cm)^1^** | **Margin** | **Growth rate** | **Texture** |  |
| --- | --- | --- | --- | --- | --- | --- | --- | --- | --- |
|  |  |  |  |  |  |  |  |  |  |
| M4A3 Oatmeal | MEA | 25 | Dark gray in the center, white in the edge | Cream | 8.67 ± 0,.29 | Entire | Fast | Cottony |  |
|  |  | 37 | Dark gray | Cream | 9.00 ± 0.00 | Entire | Fast | Cottony |  |
|  |  | 45 | There was no growth | | | | | |  |
|  | PDA | 25 | Dark gray in the center, white in the edge | White | 4.90 ± 0.10 | Entire | Fast | Cottony |  |
|  |  | 37 | Dark gray | White | 7.55 ± 0.10 | Entire | Fast | Cottony |  |
|  |  | 45 | There was no growth | | | | | |  |
|  | OA | 25 | Light gray in the center, white in the edge | White | 5.40 ± 0.10 | Entire | Fast | Cottony |  |
|  |  | 37 | Dark gray | White | 7.40 ± 0.10 | Entire | Fast | Cottony |  |
|  |  | 45 | There was no growth | | | | | |  |
| M4A4 Oatmeal | MEA | 25 | Dark gray en el centro, Light grey en los bordes | Cream | 7.60 ± 0.10 | wavy | Medium | Cottony |  |
|  |  | 37 | Dark gray | Cream | 8.90 ± 0.00 | Entire | Fast | Cottony |  |
|  |  | 45 | There was no growth | | | | | |  |
|  | PDA | 25 | Dark gray en el centro, Light grey en el borde | White | 6.53 ± 0.24 | wavy | Medium | Cottony |  |
|  |  | 37 | Dark gray | White | 9.00 ± 0.00 | Entire | Fast | Cottony |  |
|  |  | 45 | There was no growth | | | | | |  |
|  | OA | 25 | Light gray in the center, white in the edge | White | 5.53 ± 0.15 | Entire | Slow | Cottony |  |
|  |  | 37 | Light gray in the center, white in the edge | White | 7.25 ± 0.19 | Entire | Slow | Cottony |  |
|  |  |  |  |  |  |  |  |  |  |
|  |  | 45 | There was no growth | | | | | |  |
